# Supplementary material for: The NSP3 protein of SARS-CoV-2 binds fragile X mental retardation proteins to disrupt UBAP2L interactions
Source: EMBO Rep. 2024 Jan 2;25(2):25. doi: 10.1038/s44319-023-00043-z (PMC10897489; doi:10.1038/s44319-023-00043-z)
Supplement: Supplementary file 1 — Appendix [file 44319_2023_43_MOESM1_ESM.pdf]

Appendix files Garvanska et al.

Table of content

Page 2: Appendix Figure S1

Page 3-4: Appendix Figure S2

Page 5: Appendix Table S1

Page 6-16: Appendix Table S2

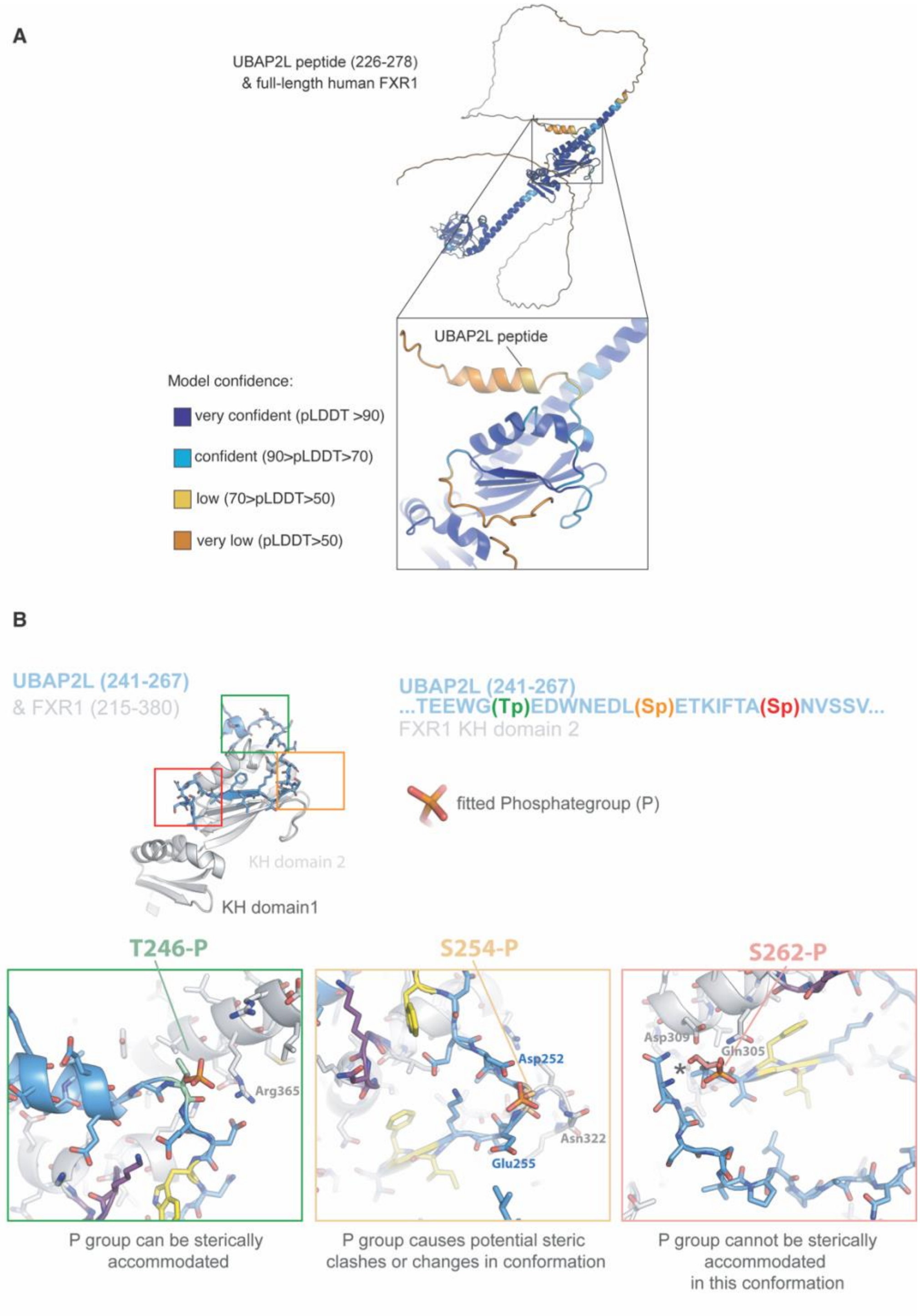

Appendix Figure S1. Model of interaction of FXR1 to UBAP2L.

A) Confidence plots of AlphaFold model of UBAP2L peptide 226-278 binding to full length FXR1.

B) Reported phosphorylation sites in UBAP2L were fitted into the AlphaFold of the FXR1-UBAP2L complex.

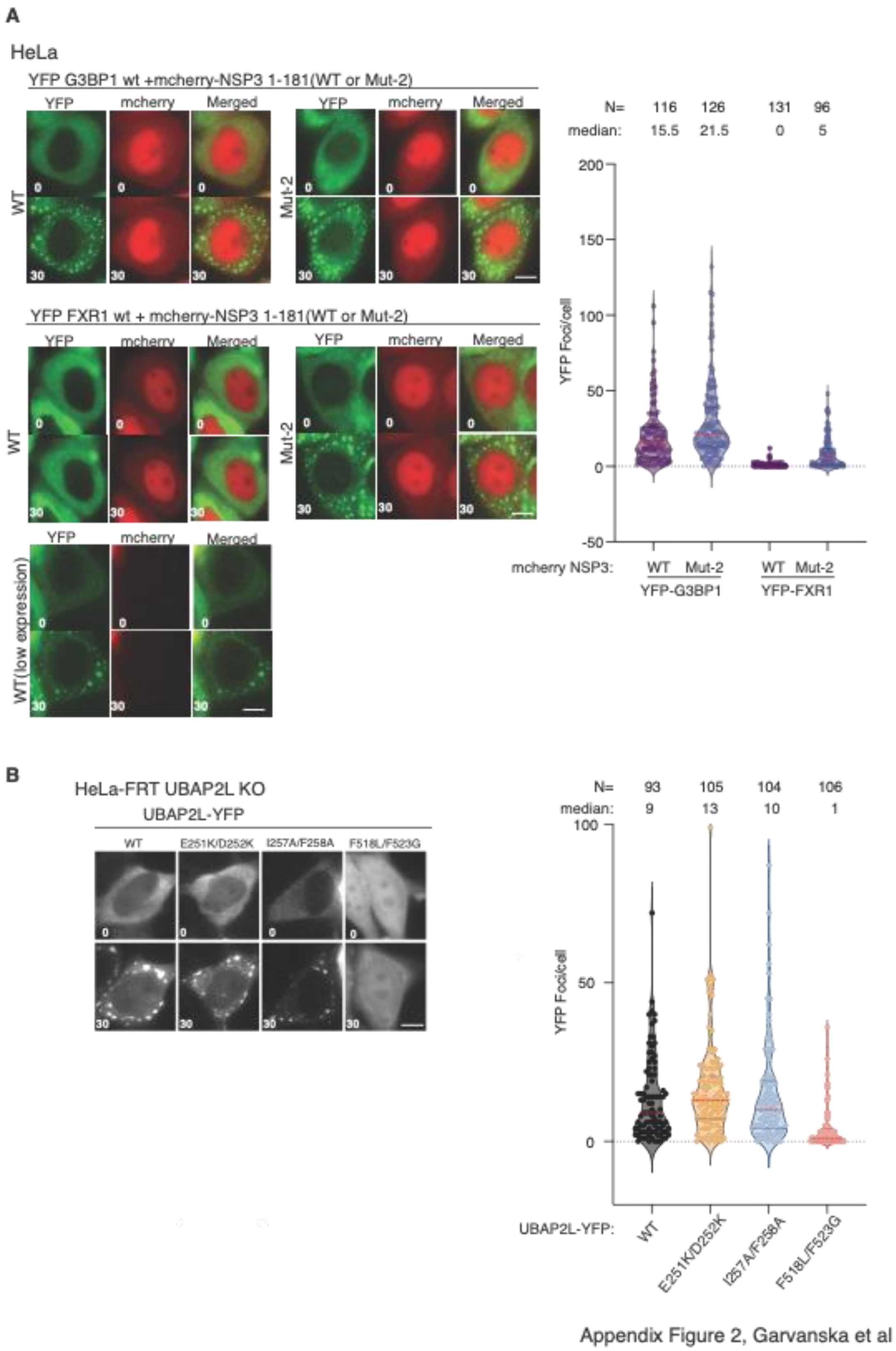

Appendix Figure S2. Analysis of stress granule components by live cell microscopy

A) The indicated YFP tagged stress granule proteins were expressed in HeLa cells in the presence of mCherry tagged NSP3 constructs and monitored by live cell microscopy. Stress granule formation was induced by arsenite and number of stress granules determined after 30 min. A pool of 3 biological replicates is shown in the graph.

B) The indicated UBAP2L-YFP constructs were expressed in HeLa-FRT UBAP2L KO cells and monitored by live cell microscopy. Arsenite was added and localization determined after 30 minutes. In A-B representative stills from the immunofluorescence is shown. The combined data from 2 biological replicates is shown in the graphs.

Data information: Violin plots with the median indicated with red line. The median and number of cells (N) analyzed per condition is indicated above the plot. Scale bar of 10 μm indicated in lower right corner.

NSP3 primers:
NSP3 FW BglII GTCAAGATCTATGGCCCCTACCAAGGTG
NSP3 Rev NotI GTCAGCGGCCGCTTACTTGCTGAAGTTTGGAG
NSP3 995 rev GTACGCGGCCGCTCAATCATCTAACCAATCTTCTTC
NSP3 996 For GTACGGATCCGATAGTCAACAAACTGTTGG
NSP3 1324 Rev GTACGCGGCCGCTCATTCAGTAGTGCCACCAGCC
NSP3 1325 Fw GTACGGATCCATGCTAGCGAAAGCTTTGAG
NSP3 1875 Rev GTACGCGGCCGCTCAAGGACCTTTGTATTCTGAGG
NSP3 1876 FW GTACGGATCCATTACGGATGTTTTCTACAAAG

FXR1 primers:
FXR1 FW BamHI GTAC GGATCC ATGGCGGAGCTGACGGTGG
FXR1 Rev 118 GTAC GCGGCCGC TCAATTTTGATTGACAGGCCG
FXR1 Rev 215 GTAC GCGGCCGC TCAAAGTTGTTTTGTGCATTC
FXR1 Rev 360 GTAC GCGGCCGC TCATTCCTTTAGATAGGCAATATG
FXR1 Rev 460 GTAC GCGGCCGC TCAACCACGTGGTCCACCACGAC
FXR1 Rev FL GTAC GCGGCCGC TCAATCACATCTTTTGCCTAG
FXR1_I304/N_Frw GGAAAAAATGGCAAAGTTAATCAAGAAATAGTGG
FxR1_I304/N_Rev CCACTATTTCTTGATTAACTTTGCCATTTTTTCC
FXR1 L351P FW GTGCAGGTTCTTCCAGAGTATCATATTGCC
FXR1 L351P Rev GGCAATATGATACTCTGGAAGAACCTGCAC

UBAP2L primers:
UBAP2L_Frw GATCGCGGCCGCATGATGACATCGGTGGGCACTAACC
UBAP2L_200_rev CATGGCGGCCGCTTAGTTAAAGGTTCCCATTCC
UBAP2L_400_rev CATGGCGGCCGCTTATGATGGGGATTGTGTCGTCG
UBAP2L_600_rev CATGGCGGCCGCTTACCGAGTCTGTGTGGAT
UBAP2L_800_rev CATGGCGGCCGCTTAAGGCAACAACGGCGGGAC
UBAP2L_FL_rev GATCGCGGCCGCTAGCTAGCGGAACCGTTGCC
UBAP2L_Fw_BamHI GATCGGATCCATGATGTCGGTGGGCACTAACC
UBAP2L_225_rev CATGGCGGCCGCTTAAGTGTTGTTCCACGTATTGC
UBAP2L_250_rev CATGGCGGCCGCTTAATTCCAATCTTCAGTCCC
UBAP2L_275_rev CATGGCGGCCGCTTATGTCACATTCTCCGCAGG
UBAP2L_300_rev CATGGCGGCCGCTTAAGATGAATCATTCTCC
UBAP2L_325_rev CATGGCGGCCGCTTATGATATGGCAGTCTGC
UBAP2L_350_rev CATGGCGGCCGCTTAACCGACATCACCAAATCC
UBAP2L_375_rev CATGGCGGCCGCTTACAACTGAGCCAGGGC
UBAP2L EE251-252KK F CTGAAGATTGGAATAAGAAGCTTTCTGAGACCAAG
UBAP2L EE251-252KK R CTTGGTCTCAGAAAGCTTCTTATTCCAATCTTCAG
UBAP2L I257A/F258A_FW: CTT TCT GAG ACC AAG GCC GCC ACT GCC TCT AAT GTG
UBAP2L I257A/F258A _Rev: CAC ATT AGA GGC AGT GGC GGC CTT GGT CTC AGA AAG

Appendix Table S1.

Overview of primers used to clone NSP3, FXR1 and UBAP2L constructs.

Appendix Table S2. Affinities and thermodynamic values of NSP3 1-181 binding events inferred from ITC measurements performed at 25 °C. Gibbs free energy (ΔG), enthalpy (ΔH), entropy (-TΔS), equilibrium dissociation constant (K_D) and reaction stoichiometry (n) are shown. The protein-protein interaction affinity is defined by the Gibbs energy for binding ΔG = -RT lnK_A = RT lnK_D.

	**ΔG (*kcal mol^{-1}*)**	**ΔH (*kcal mol^{-1}*)**	**-TΔS (*kcal mol^{-1}*)**	**K_D (*μM*)**	***n***
NSP3 1-181/FXR1$^{1-122}$			*No binding*		
NSP3 1-181/FXR1$^{212-289}$			*No binding*		
NSP3 1-181/FXR1$^{215-360}$	-7.70	-25.9 ± 0.38	18.2	2.26 ± 0.13	0.90 ± 0.01
NSP3 1-181mut1/FXR1$^{215-360}$			*No binding*		
NSP3 1-181/FXR1$^{215-360}$ I304N			*No binding*		

Appendix Table S2 continued. Affinities and thermodynamic values of FXR1$^{215\text{-}360}$ binding events inferred from ITC measurements performed at 25 °C. Gibbs free energy (ΔG), enthalpy (ΔH), entropy (-TΔS), equilibrium dissociation constant (K_D) and reaction stoichiometry (n) are shown. The protein-protein interaction affinity is defined by the Gibbs energy for binding $\Delta G = -RT \ln K_A = RT \ln K_D$. [a]The stoichiometry (n) was fixed to 1 when not explicitly stated.

FXR1$^{215\text{-}360}$ complex	**ΔG (*kcal mol*$^{-1}$)**	**ΔH (*kcal mol*$^{-1}$)**	**-TΔS (*kcal mol*$^{-1}$)**	**K_D (*μM*)**	***n***
FXR1$^{215\text{-}360}$/NSP3 WT peptide	-7.86	-24.20 ± 0.12	16.3	1.74 ± 0.04	0.95 ± 0.01
FXR1$^{215\text{-}360}$/NSP3 M1	-5.49	-18.40 ± 8.67	13.0	95.1 ± 43.2	
FXR1$^{215\text{-}360}$/NSP3 M2			*No binding*		
FXR1$^{215\text{-}360}$/NSP3 M3			*No binding*		
FXR1$^{215\text{-}360}$/NSP3 M4			*No binding*		

Appendix Table S2 continued**.** Affinities and thermodynamic values of NSP3 alphavirus binding events inferred from ITC measurements performed at 25 °C. Gibbs free energy (ΔG), enthalpy (ΔH), entropy (-TΔS), equilibrium dissociation constant (K_D) and reaction stoichiometry (n) are shown. The protein-protein interaction affinity is defined by the Gibbs energy for binding $\Delta G = -RT \ln K_A = RT \ln K_D$. [a]The stoichiometry (n) was fixed to 1 when not explicitly stated.

	**ΔG (*kcal mol^{-1}*)**	**ΔH (*kcal mol^{-1}*)**	**-TΔS (*kcal mol^{-1}*)**	**K_D (*μM*)**	***^{a}n***
NSP3 peptide alphavirus/FXR1$^{1\text{-}122}$	-6.43	-6.11 ± 0.65	-0.32	19.4 ± 3.16	
NSP3 peptide alphavirus/FXR1$^{212\text{-}289}$			*No binding*		
NSP3 peptide alphavirus/FXR1$^{215\text{-}360}$			*No binding*		

***Appendix Table S2 continued*.** Affinities and thermodynamic values of FXR1$^{215\text{-}360}$ binding events inferred from ITC measurements performed at 25 °C. Gibbs free energy (ΔG), enthalpy (ΔH), entropy (-TΔS), equilibrium dissociation constant (K_D) and reaction stoichiometry (n) are shown. The protein-protein interaction affinity is defined by the Gibbs energy for binding $\Delta G = -RT \ln K_A = RT \ln K_D$. [a]The stoichiometry (n) was fixed to 1 when not explicitly stated.

FXR1$^{215\text{-}360}$ complex	**ΔG (*kcal mol^{-1}*)**	**ΔH (*kcal mol^{-1}*)**	**-TΔS (*kcal mol^{-1}*)**	**K_D (*μM*)**	***n***
FXR1$^{215\text{-}360}$/UBAP2L$^{263\text{-}290}$	-6.92	-13.7 ± 0.54	6.79	8.53 ± 0.69	0.96 ± 0.01
FXR1$^{215\text{-}360}$/UBAP2L$^{263\text{-}290}$ *pS274*	-6.27	-8.91 ± 2.38	2.65	25.5 ± 7.34	0.92 ± 0.01
FXR1$^{215\text{-}360}$/UBAP2L$^{263\text{-}290}$ *pT266*	-7.42	-12.0 ± 0.26	4.57	3.66 ± 0.24	0.90 ± 0.01
FXR1$^{215\text{-}360}$/UBAP2L$^{263\text{-}290}$ *pS282*			*No binding*		

***Appendix Table S2 continued*.** Affinities and thermodynamic values of NF1/N protein from SARS-CoV2 and FXR1 215-360 binding inferred from ITC measurements performed at 25 °C. Gibbs free energy (ΔG), enthalpy (ΔH), entropy (-TΔS), equilibrium dissociation constant (K_D) and reaction stoichiometry (n) are shown. The protein-protein interaction affinity is defined by the Gibbs energy for binding $\Delta G = -RT \ln K_A = RT \ln K_D$. [a]The stoichiometry (n) was fixed to 1 when not explicitly stated.

FXR1$^{215\text{-}360}$ complex	**ΔG (*kcal mol^{-1}*)**	**ΔH (*kcal mol^{-1}*)**	**-TΔS (*kcal mol^{-1}*)**	**K_D (*µM*)**	***n***
N SARS-CoV-2/NF1 RNA	-8.76	-30.1 ± 0.68	21.3	0.38 ± 0.04	0.82 ± 0.01
FXR1$^{215\text{-}360}$/NF1 RNA			*No binding*		

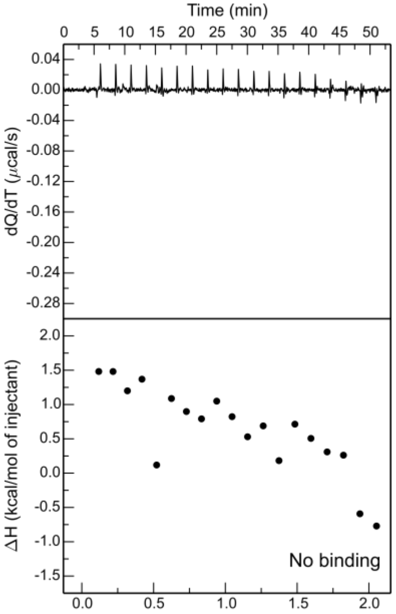

FXR1 1-122/GST-NSP3 1-181

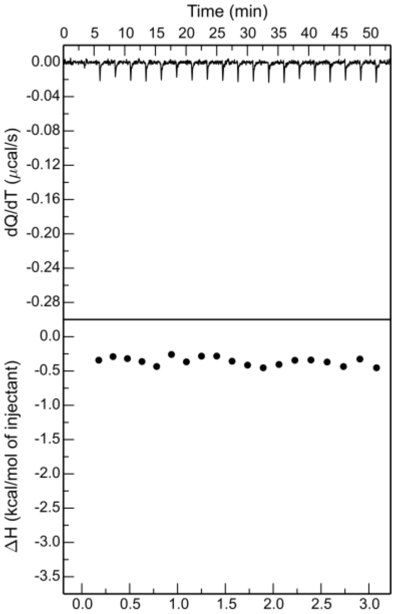

FXR1 212-289/GST-NSP3 1-181

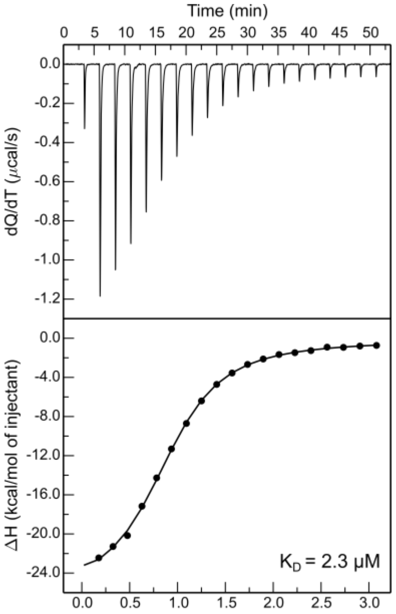

FXR1 215-360/GST-NSP3 1-181

Appendix Table S2 continued

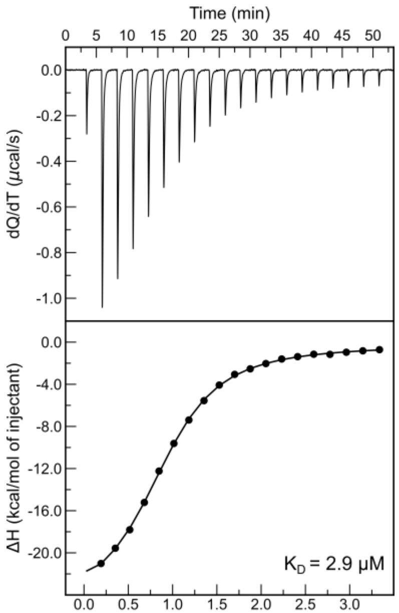

FXR1 215-360/His-NSP3 1-181 WT

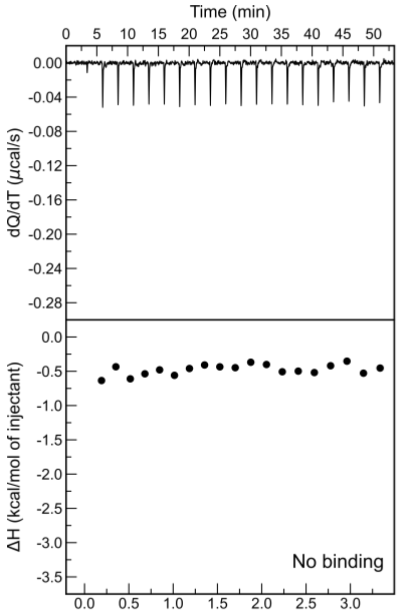

FXR1 215-360/His-NSP3 1-181 Mut1

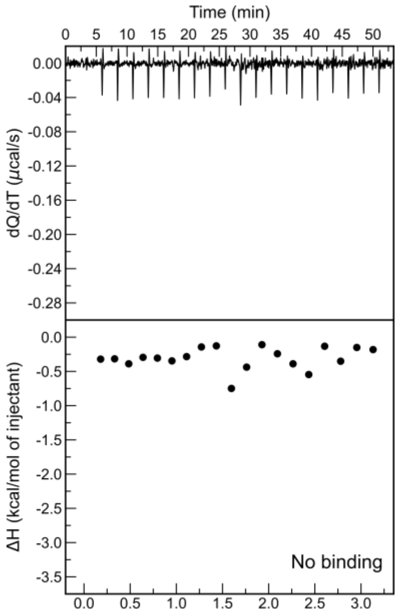

FXR1 215-360 I304N/His-NSP3 1-181

Appendix Table S2 continued

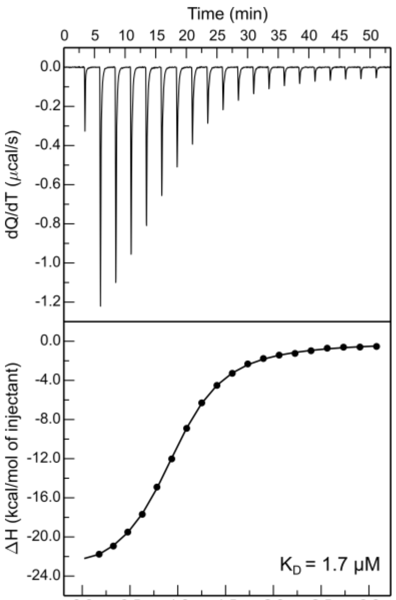

FXR1 215-360/NSP3 peptide WT

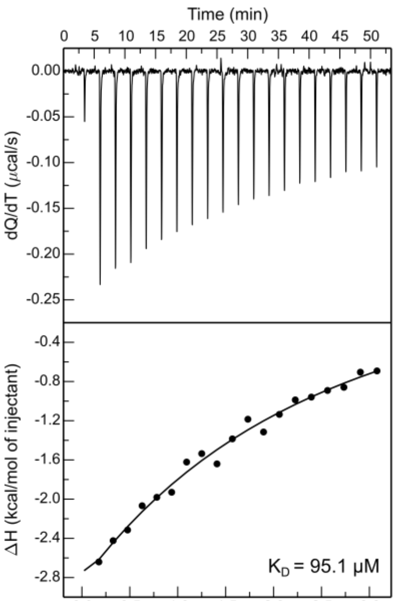

FXR1 215-360/NSP3 peptide M1

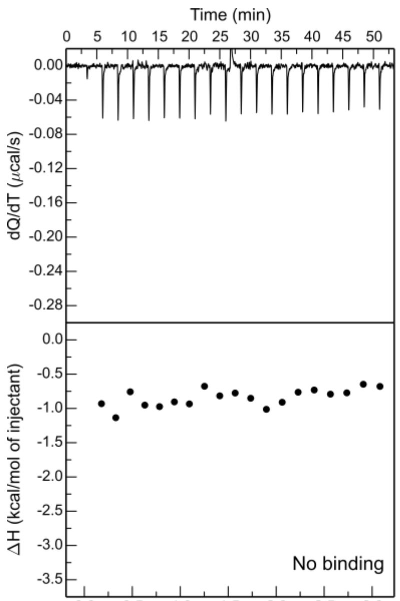

FXR1 215-360/NSP3 peptide M2

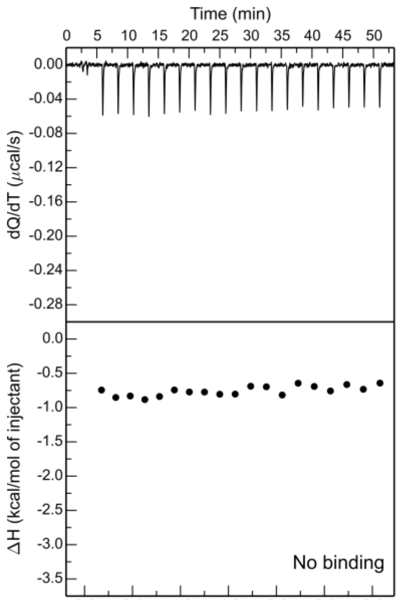

FXR1 215-360/NSP3 peptide M3

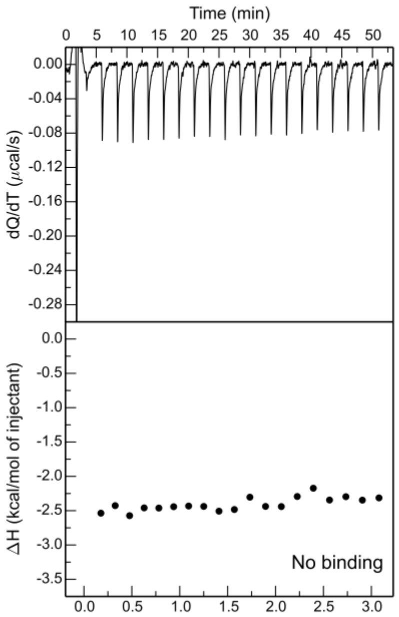

FXR1 215-360/NSP3 peptide M4

Appendix Table S2 continued

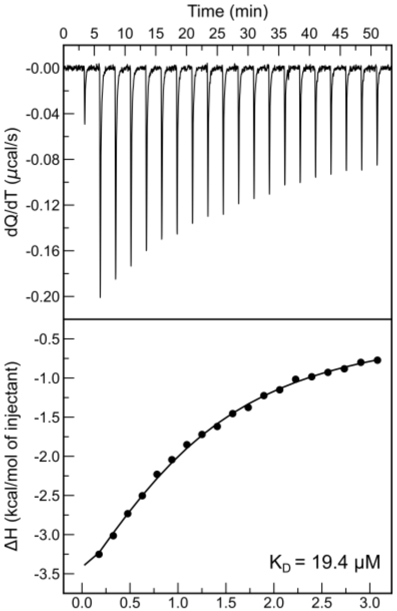

FXR1 1-122/NSP3 peptide alphavirus

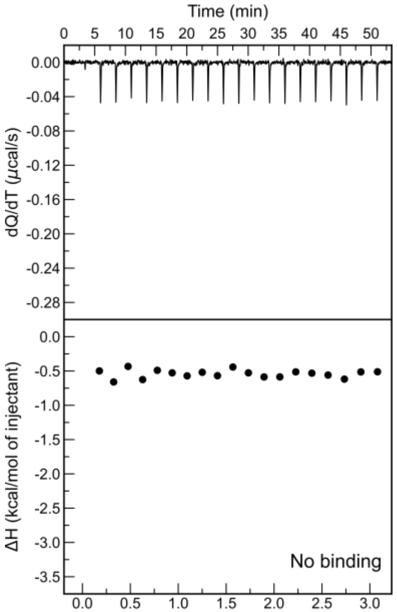

FXR1 212-289/NSP3 peptide alphavirus

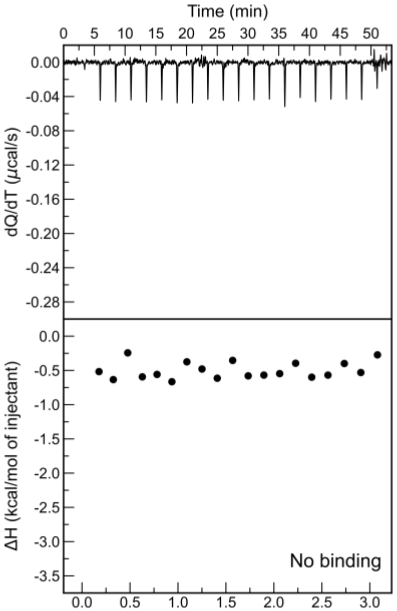

FXR1 215-360/NSP3 peptide alphavirus

Appendix Table S2 continued

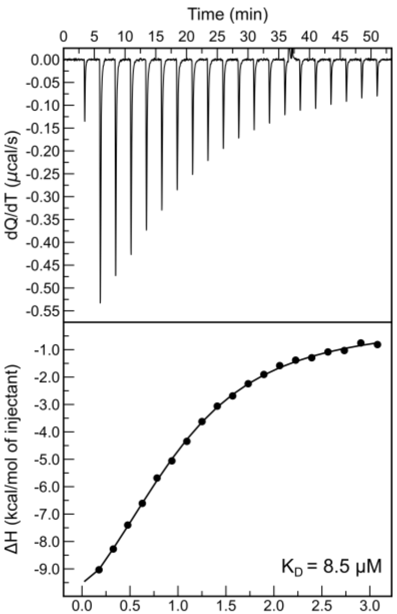

UBAP2L peptide/FXR1 215-360

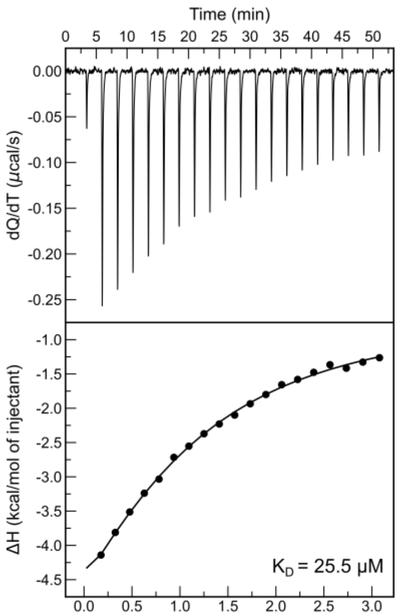

UBAP2L pS274 peptide/FXR1 215-360

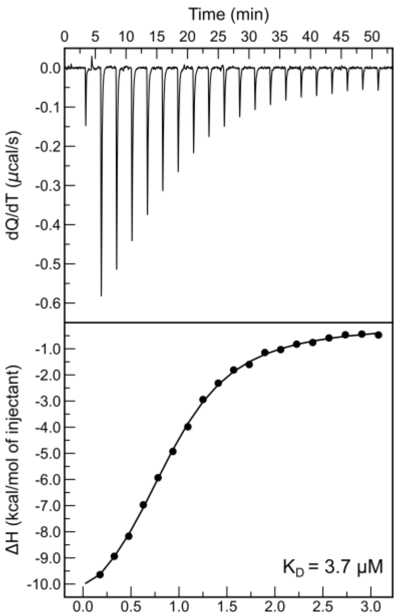

UBAP2L pT266 peptide/FXR1 215-360

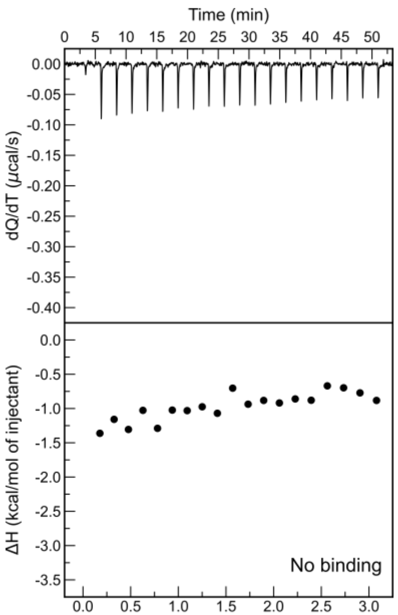

UBAP2L pS282 peptide/FXR1 215-360

Appendix Table S2 continued

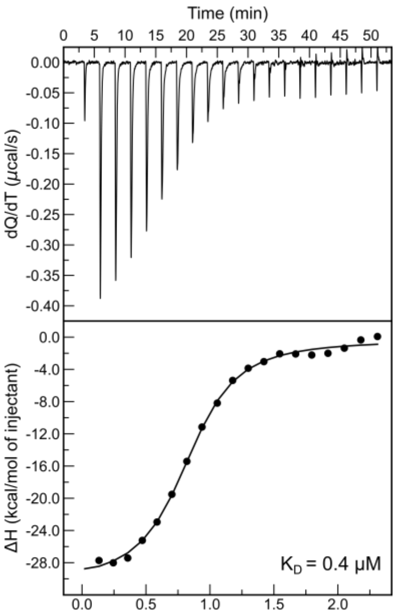

NF1 RNA/N protein SARS-CoV-2

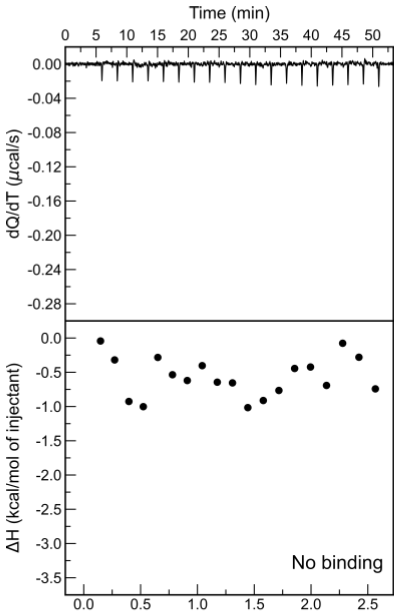

NF1 RNA/FXR1 215-360

Appendix Table S2 continued
