# Supplementary material for: The NSP3 protein of SARS-CoV-2 binds fragile X mental retardation proteins to disrupt UBAP2L interactions
Source: EMBO Rep. 2024 Jan 2;25(2):25. doi: 10.1038/s44319-023-00043-z (PMC10897489; doi:10.1038/s44319-023-00043-z)
Supplement: Supplementary file 3 — Source Data Fig. 1 [file 44319_2023_43_MOESM3_ESM.zip › Figure 1/1C/1C_ all_uncropped_WB_images.pdf]

1C
Raw images

Input

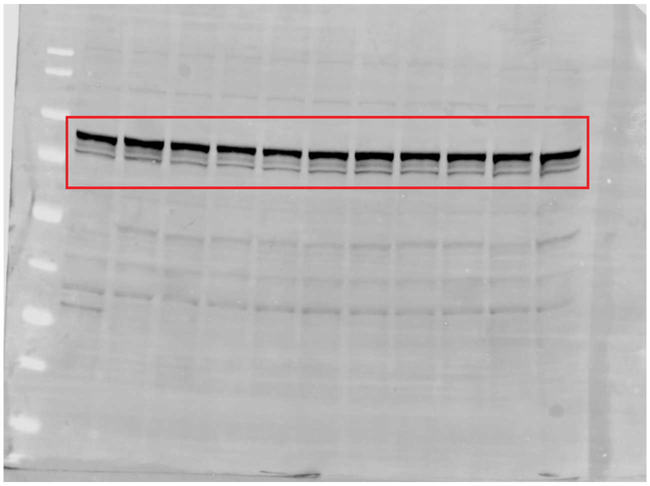

IP

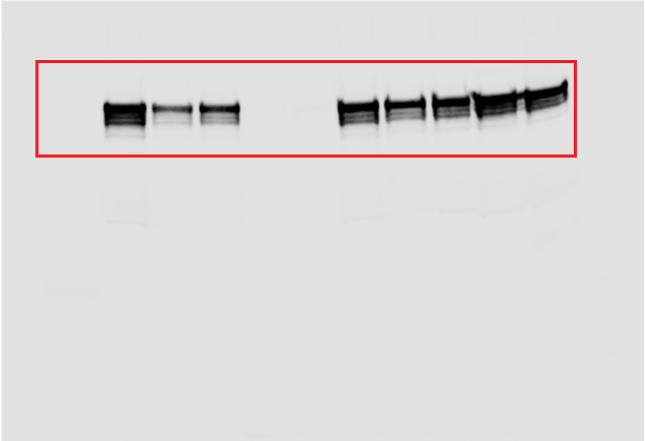

FMR
rabbit 800
Probed for 3rd

Input

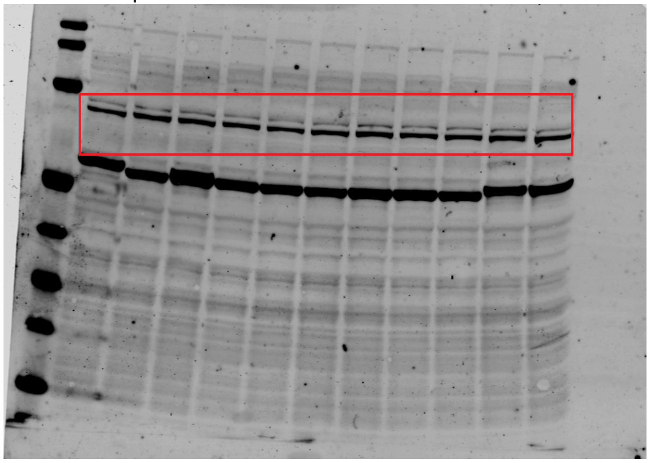

IP

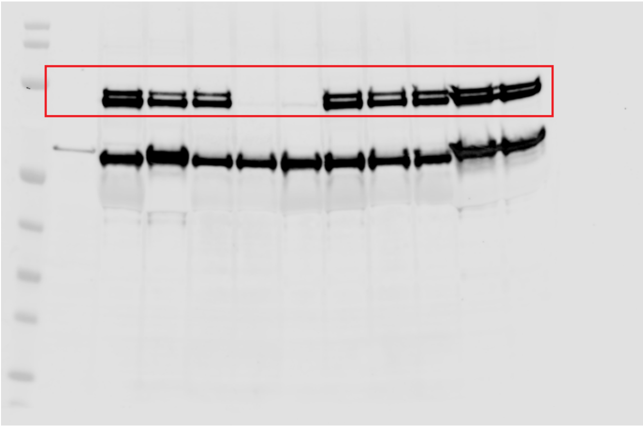

FXR1
mouse 680
Probed for 2nd

input

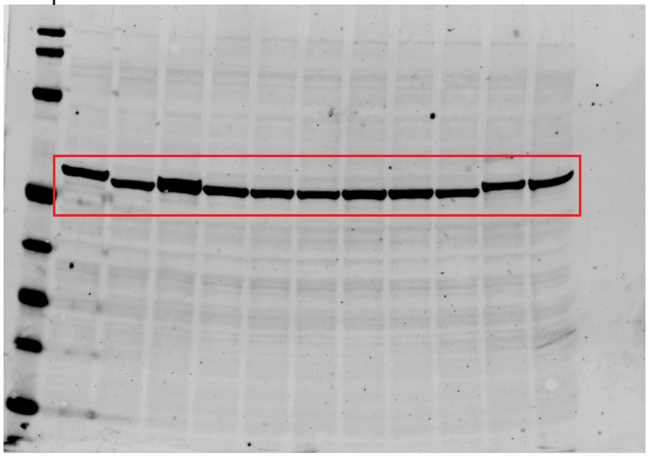

IP

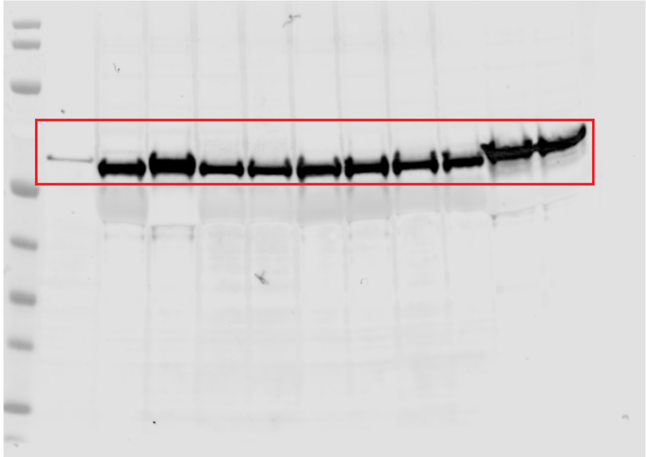

myc
mouse 680
Probed for 1st

Input

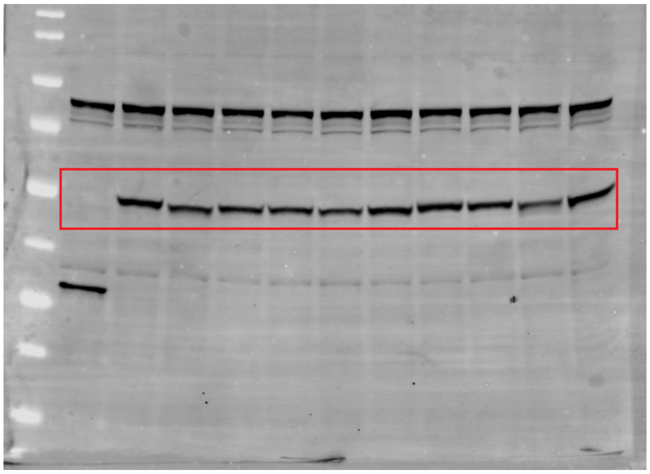

IP

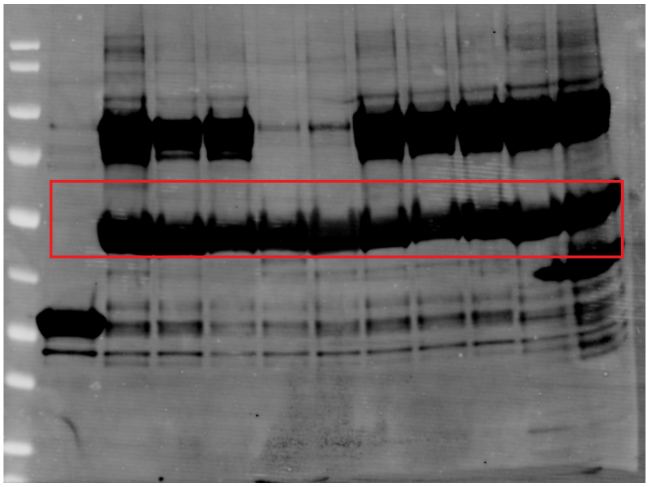

GFP
rabbit- 800
Probed for last
