# Supplementary material for: The NSP3 protein of SARS-CoV-2 binds fragile X mental retardation proteins to disrupt UBAP2L interactions
Source: EMBO Rep. 2024 Jan 2;25(2):25. doi: 10.1038/s44319-023-00043-z (PMC10897489; doi:10.1038/s44319-023-00043-z)
Supplement: Supplementary file 3 — Source Data Fig. 1 [file 44319_2023_43_MOESM3_ESM.zip › Figure 1/1D/1D.rtf]

Vero E6 cells or Calu3 cells were infected with the indicated SARS-CoV-2 viruses and viral titers measured at 24 and 48 hours post infection (n=6) 
