# Supplementary material for: The NSP3 protein of SARS-CoV-2 binds fragile X mental retardation proteins to disrupt UBAP2L interactions
Source: EMBO Rep. 2024 Jan 2;25(2):25. doi: 10.1038/s44319-023-00043-z (PMC10897489; doi:10.1038/s44319-023-00043-z)
Supplement: Supplementary file 3 — Source Data Fig. 1 [file 44319_2023_43_MOESM3_ESM.zip › Figure 1/1F/1F.rtf]

1FVero E6 cells were pretreated with control (solid) or 100 unit of type I IFN (hashed) for 16 hours and then infected with the indicated SARS-CoV-2 viruses and viral titers measured after 48 hours (n=6). Statistical analysis measured by two-tailed Student’s t-test: ***p<0.001, *p<0.05.
