# Supplementary figures and images for: The NSP3 protein of SARS-CoV-2 binds fragile X mental retardation proteins to disrupt UBAP2L interactions
Source: EMBO Rep. 2024 Jan 2;25(2):25. doi: 10.1038/s44319-023-00043-z (PMC10897489; doi:10.1038/s44319-023-00043-z)

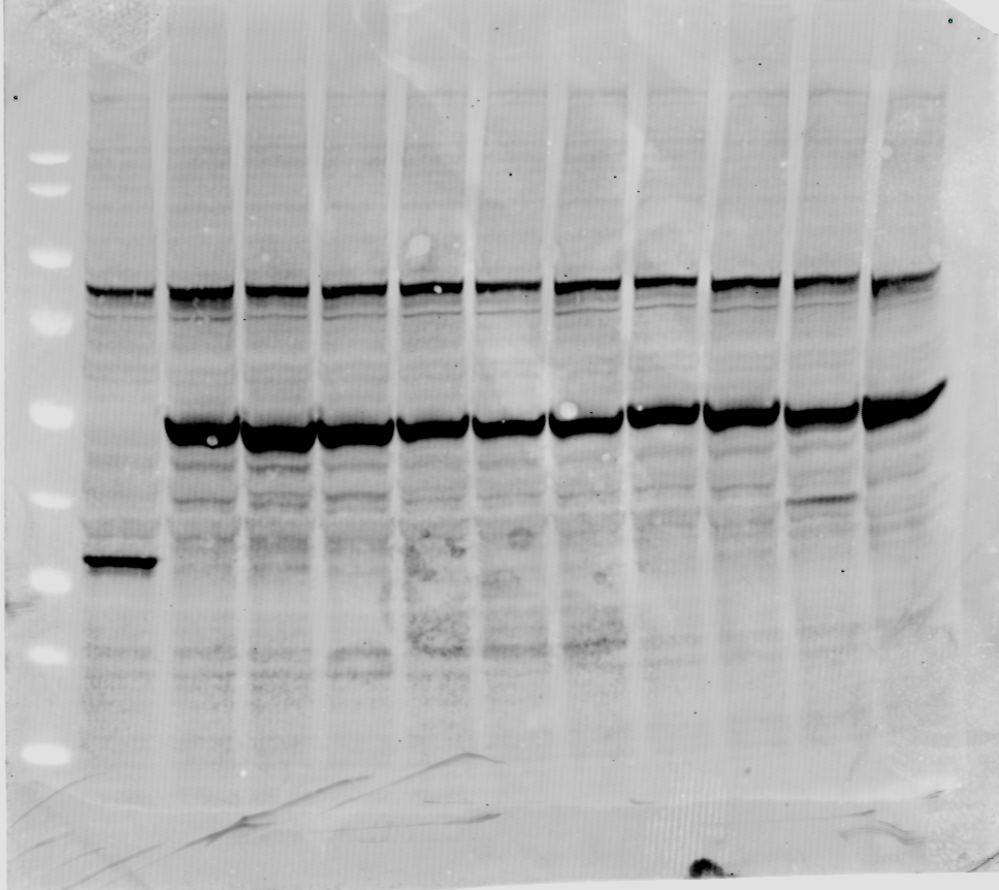

Supplement: Supplementary file 3 — Source Data Fig. 1 [file 44319_2023_43_MOESM3_ESM.zip › Figure 1/1C/ala1-fmr1-in.png]

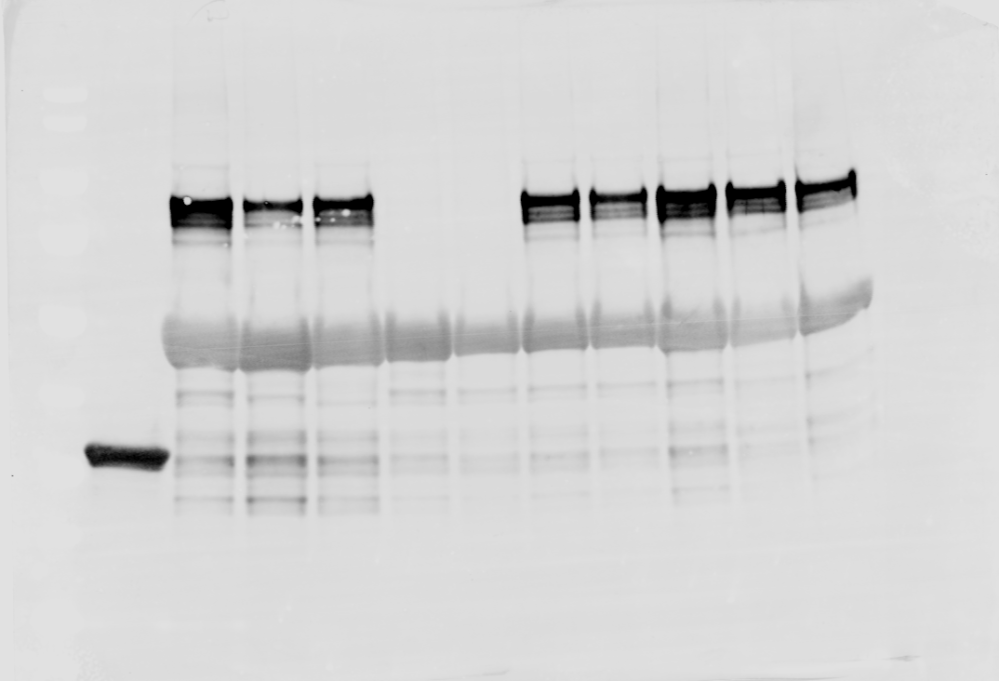

Supplement: Supplementary file 3 — Source Data Fig. 1 [file 44319_2023_43_MOESM3_ESM.zip › Figure 1/1C/ala1-fmr1-IP.png]

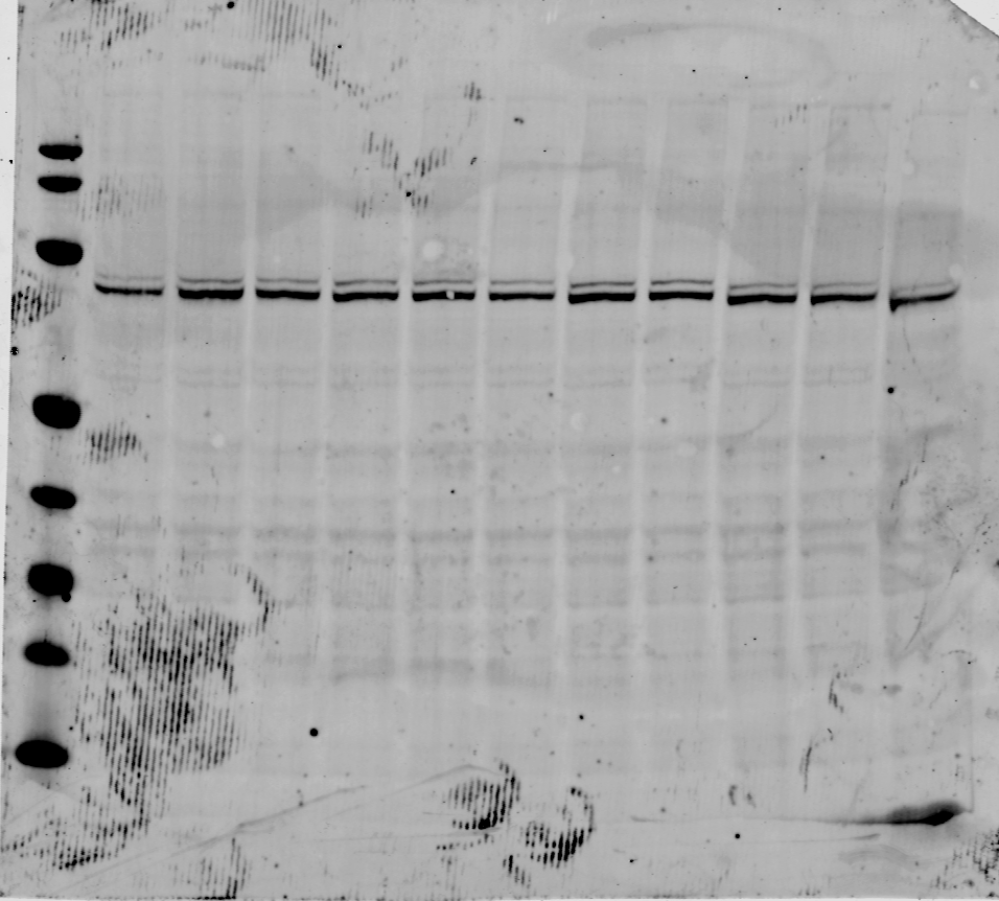

Supplement: Supplementary file 3 — Source Data Fig. 1 [file 44319_2023_43_MOESM3_ESM.zip › Figure 1/1C/ala1-fxr1-in.png]

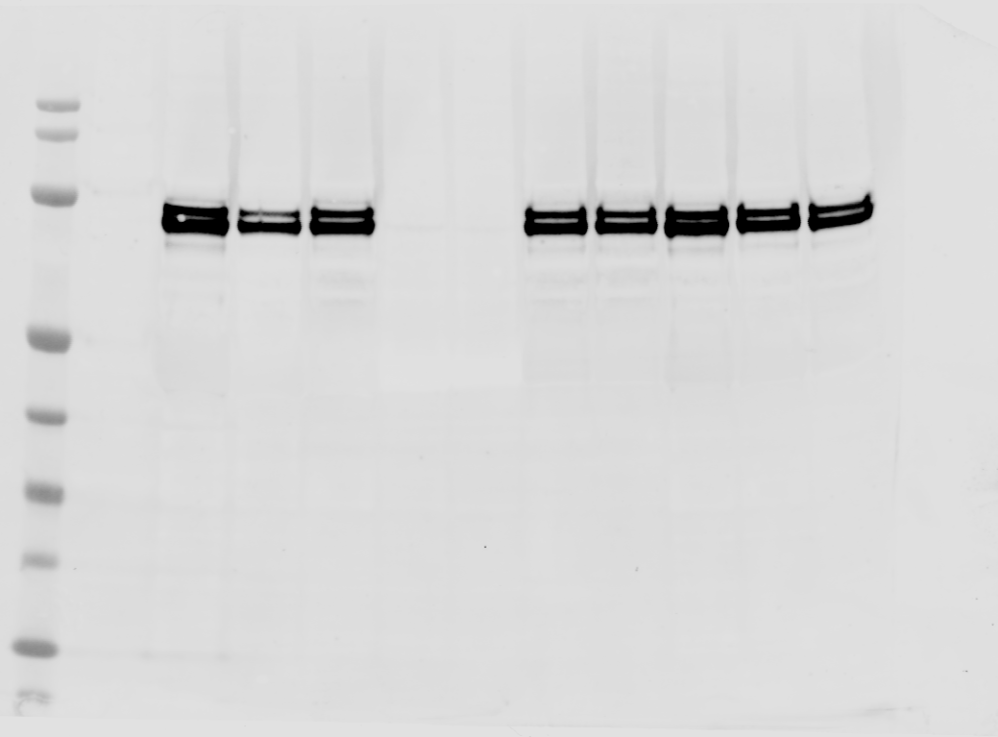

Supplement: Supplementary file 3 — Source Data Fig. 1 [file 44319_2023_43_MOESM3_ESM.zip › Figure 1/1C/ala1-fxr1-IP.png]

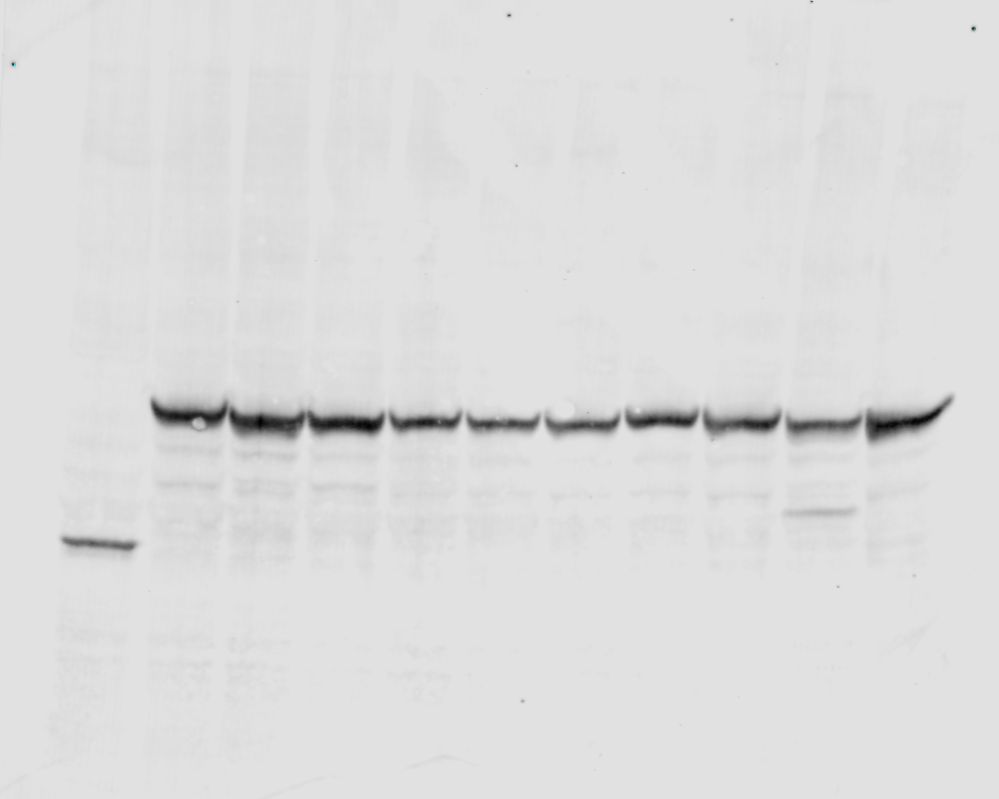

Supplement: Supplementary file 3 — Source Data Fig. 1 [file 44319_2023_43_MOESM3_ESM.zip › Figure 1/1C/ala1-gfp-in.png]

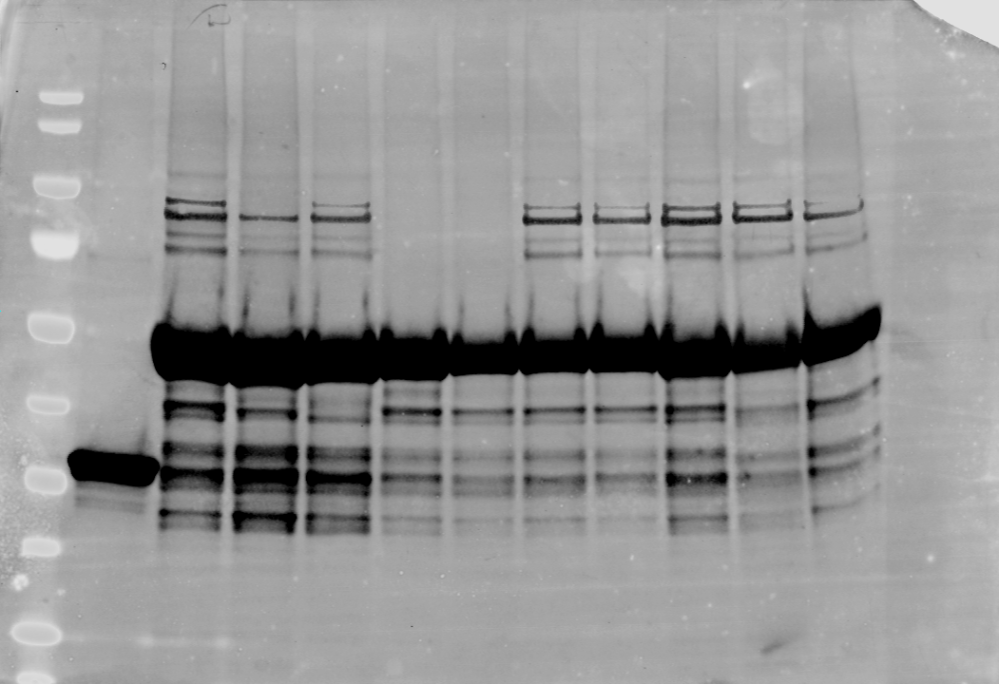

Supplement: Supplementary file 3 — Source Data Fig. 1 [file 44319_2023_43_MOESM3_ESM.zip › Figure 1/1C/ala1-gfp-IP.png]

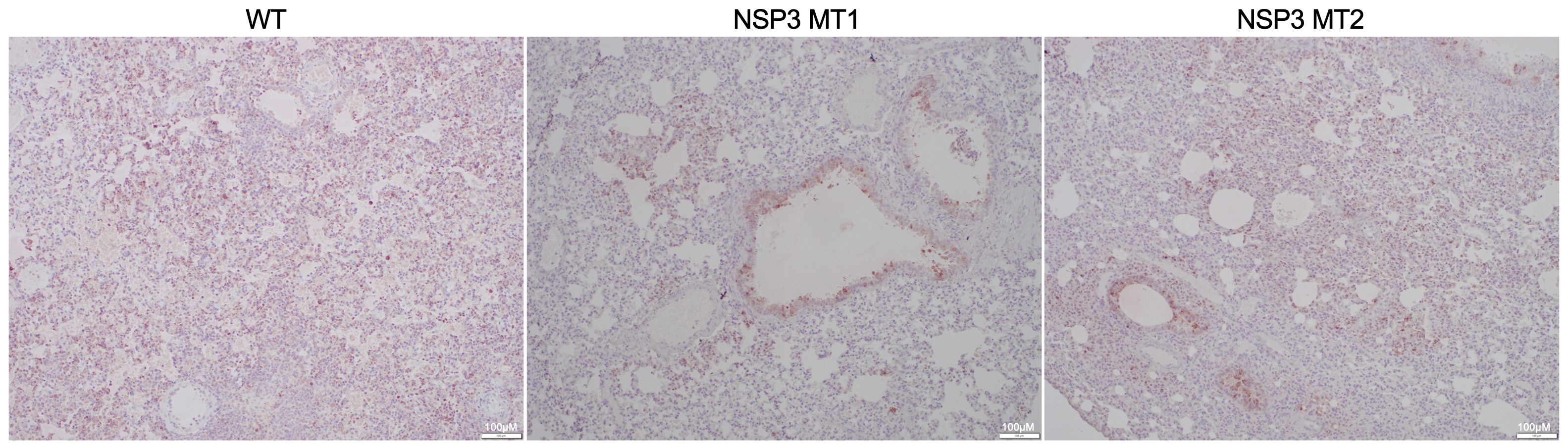

Supplement: Supplementary file 4 — Source Data Fig. 2 [file 44319_2023_43_MOESM4_ESM.zip › Figure 2/2D/NSP3 D2 Antigen stain 10x Figures.tiff]

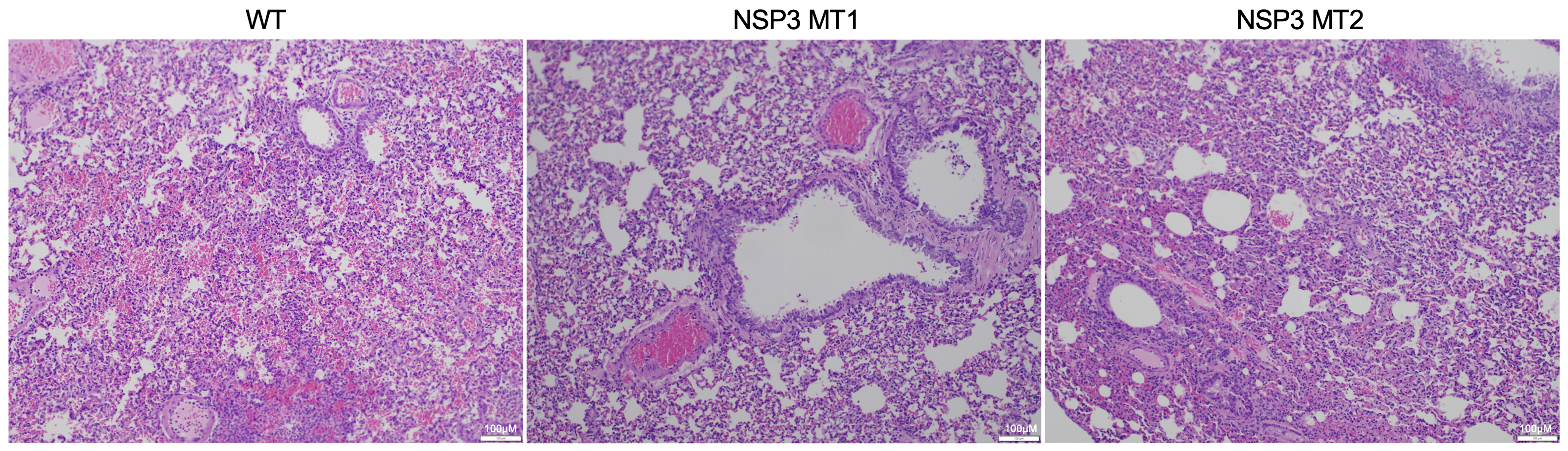

Supplement: Supplementary file 4 — Source Data Fig. 2 [file 44319_2023_43_MOESM4_ESM.zip › Figure 2/2H/NSP3 D2 Histo 10x Figures.tiff]

3A

blotted for 1st  
myc mouse  
680

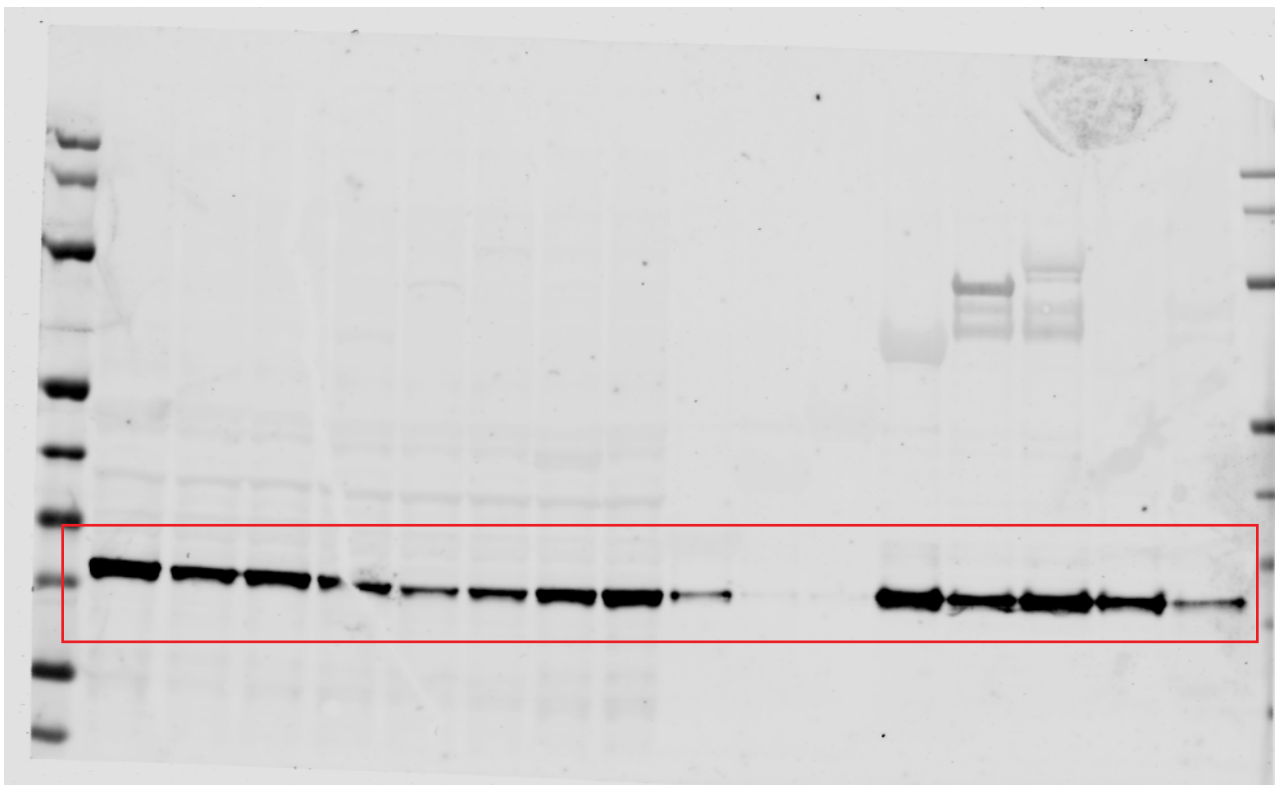

blotted for 2nd  
GFP rabbit  
800

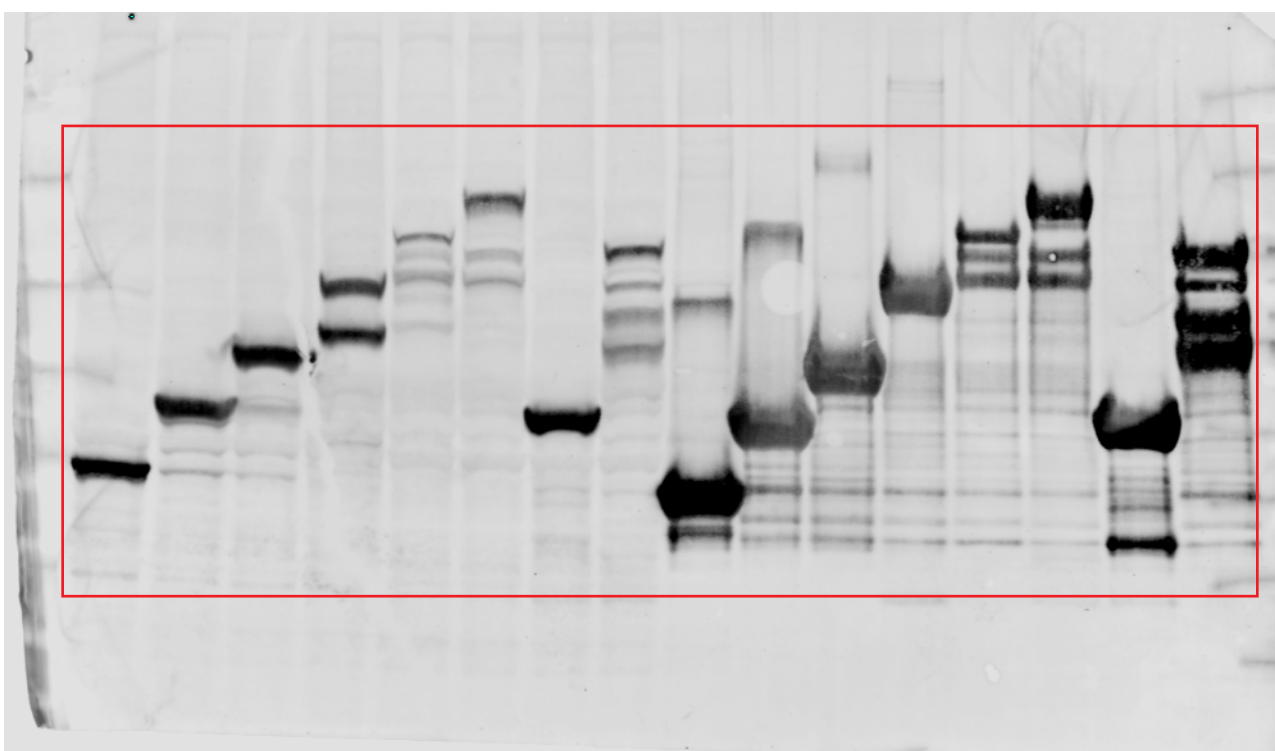

Supplement: Supplementary file 5 — Source Data Fig. 3 [file 44319_2023_43_MOESM5_ESM.zip › Figure 3/3A/3A _raw_image copy.pdf]

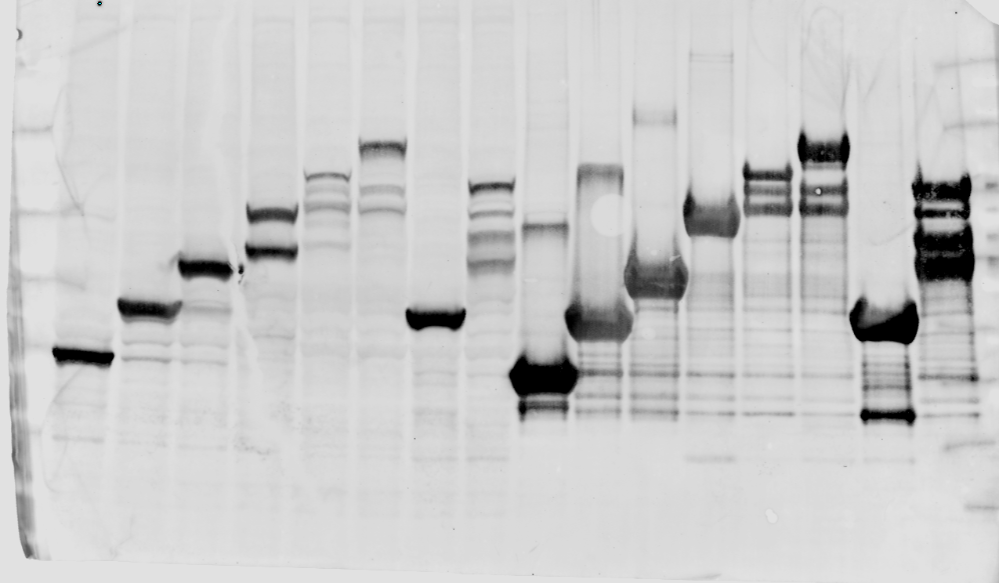

Supplement: Supplementary file 5 — Source Data Fig. 3 [file 44319_2023_43_MOESM5_ESM.zip › Figure 3/3A/fxr panel gfp.png]

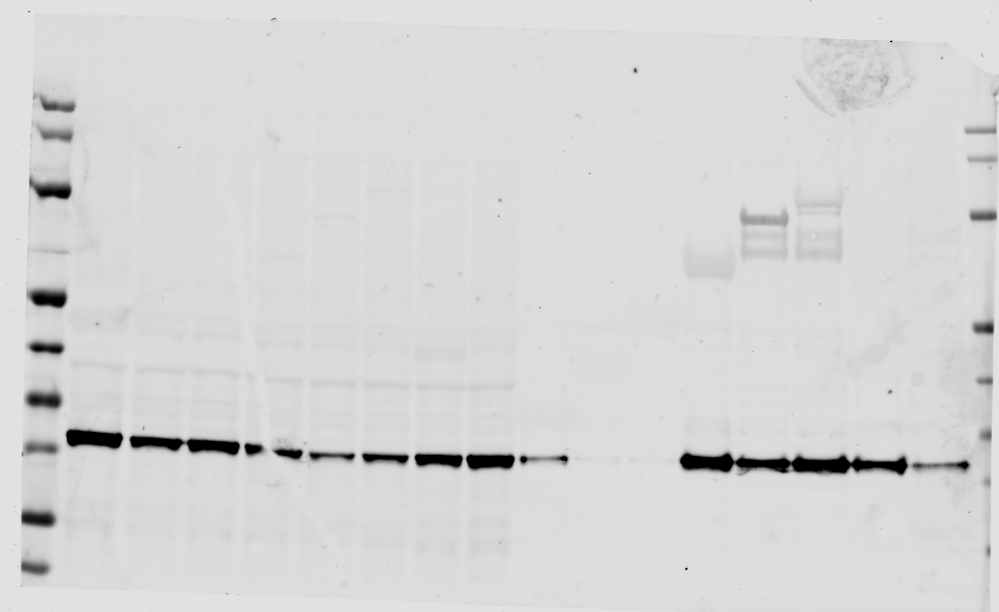

Supplement: Supplementary file 5 — Source Data Fig. 3 [file 44319_2023_43_MOESM5_ESM.zip › Figure 3/3A/fxr panel myc.png]

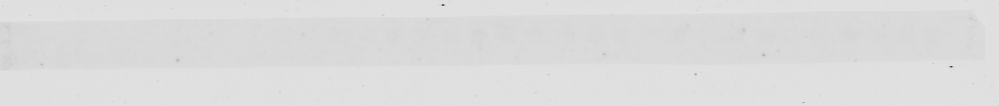

Supplement: Supplementary file 5 — Source Data Fig. 3 [file 44319_2023_43_MOESM5_ESM.zip › Figure 3/3C/nsp3pep_arrayctrl .png]

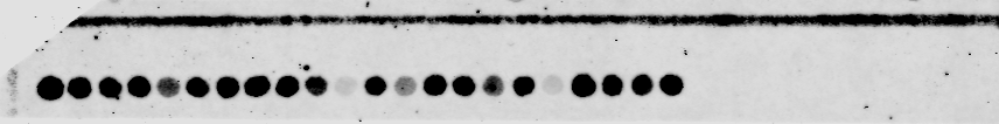

Supplement: Supplementary file 5 — Source Data Fig. 3 [file 44319_2023_43_MOESM5_ESM.zip › Figure 3/3C/peptidearray-FXR1 .png]

4C

bloted for 1st FXR1 - mouse 680

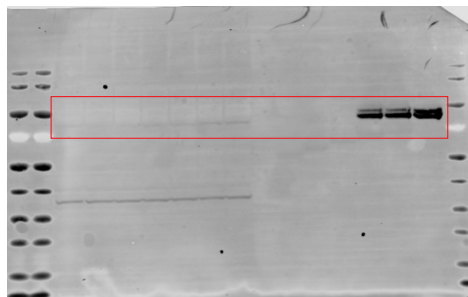

Bloted for 2nd GFP-rabbit 800

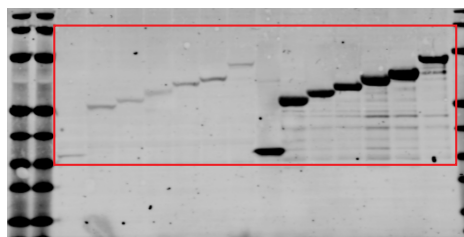

Supplement: Supplementary file 6 — Source Data Fig. 4 [file 44319_2023_43_MOESM6_ESM.zip › Figure 4/4C/4C_uncropped.pdf]

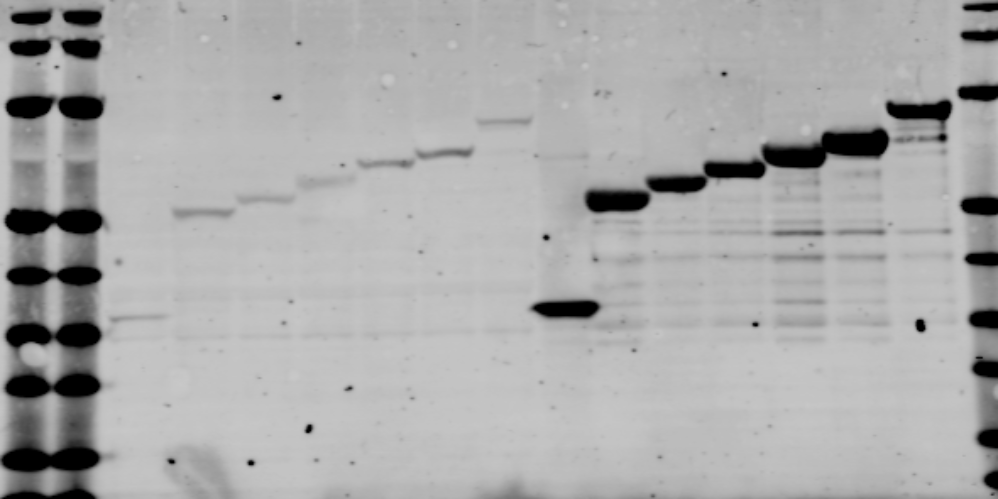

Supplement: Supplementary file 6 — Source Data Fig. 4 [file 44319_2023_43_MOESM6_ESM.zip › Figure 4/4C/ubap2l panels -700.png]

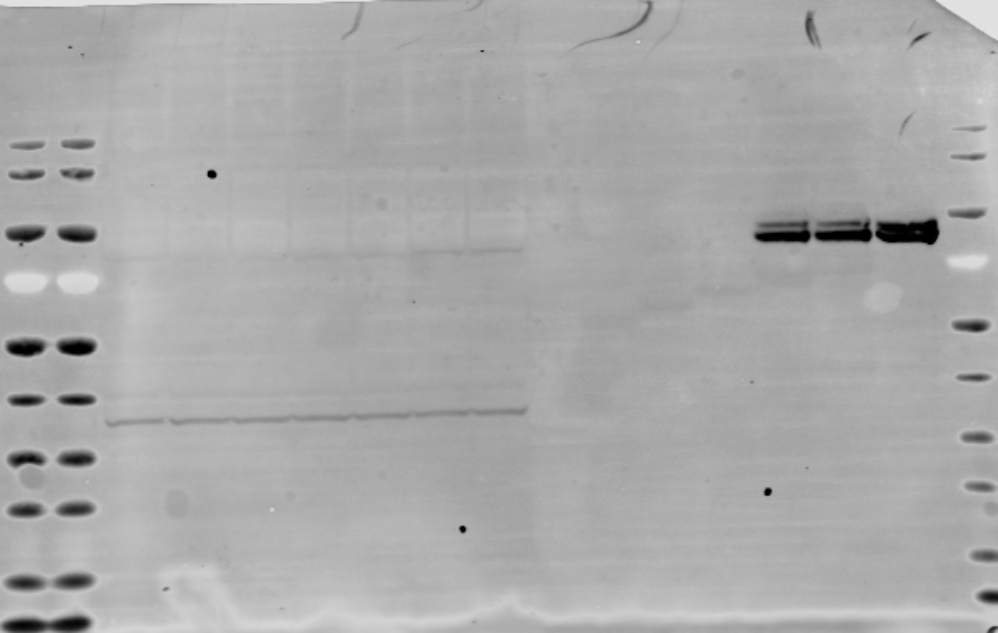

Supplement: Supplementary file 6 — Source Data Fig. 4 [file 44319_2023_43_MOESM6_ESM.zip › Figure 4/4C/ubap2l panels -800.png]

4D

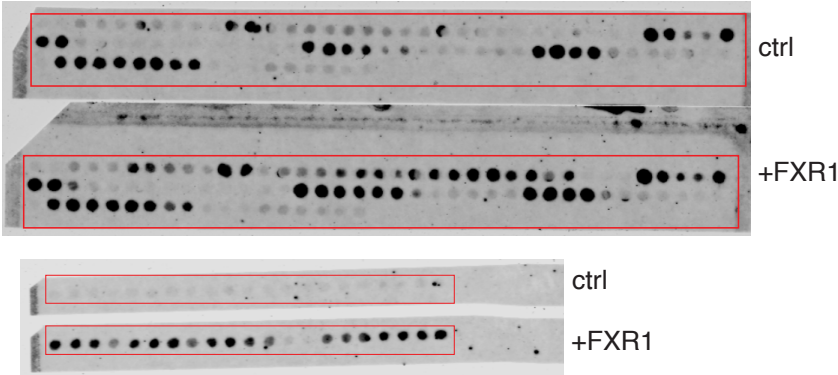

Supplement: Supplementary file 6 — Source Data Fig. 4 [file 44319_2023_43_MOESM6_ESM.zip › Figure 4/4D/4D.pdf]

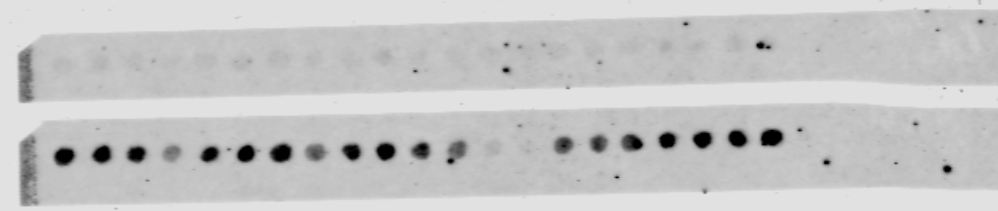

Supplement: Supplementary file 6 — Source Data Fig. 4 [file 44319_2023_43_MOESM6_ESM.zip › Figure 4/4D/UBAP2L peptide array.png]

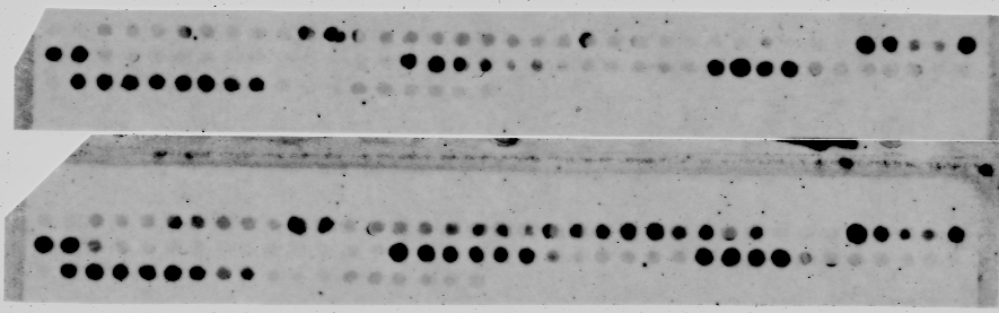

Supplement: Supplementary file 6 — Source Data Fig. 4 [file 44319_2023_43_MOESM6_ESM.zip › Figure 4/4D/UBAP2L PEPTIDEARRAY.png]

4E

bloted for last GFP rabbit 800

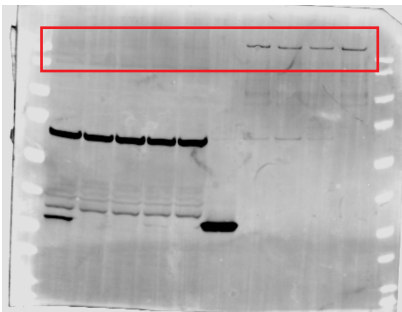

bloted for 2nd rabbit G3BP1

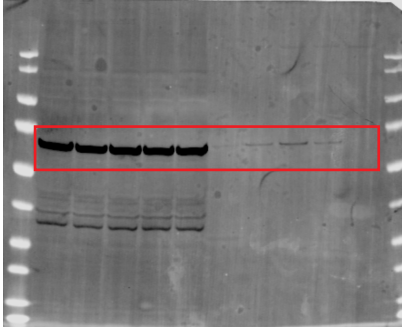

bloted for 1st FXR1 mouse 680

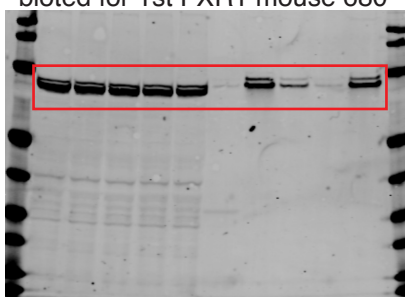

Supplement: Supplementary file 6 — Source Data Fig. 4 [file 44319_2023_43_MOESM6_ESM.zip › Figure 4/4E/4Euncropped.pdf]

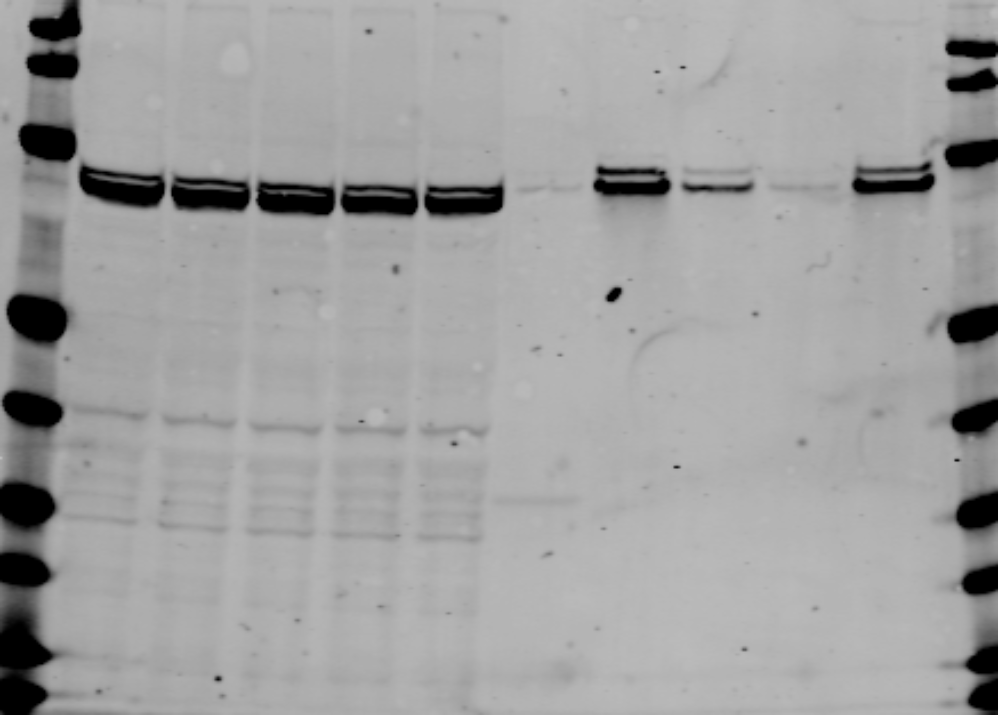

Supplement: Supplementary file 6 — Source Data Fig. 4 [file 44319_2023_43_MOESM6_ESM.zip › Figure 4/4E/UBAP2Lmutants_FXR1.png]

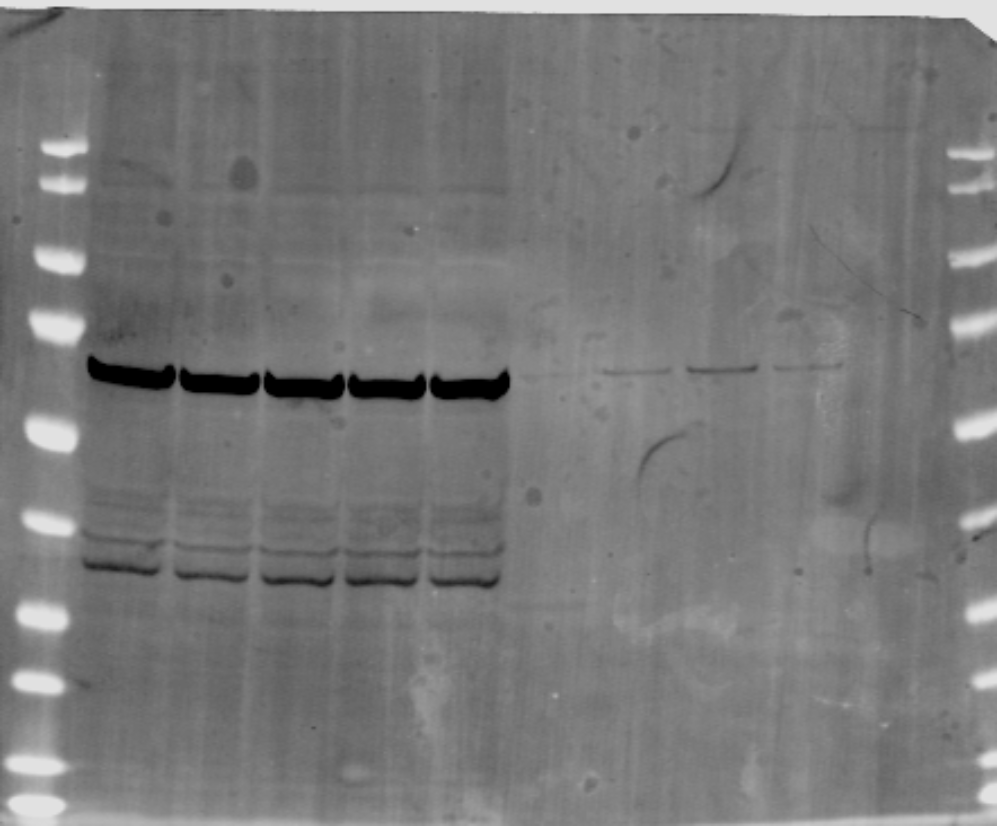

Supplement: Supplementary file 6 — Source Data Fig. 4 [file 44319_2023_43_MOESM6_ESM.zip › Figure 4/4E/UBAP2Lmutants_G3BP1.png]

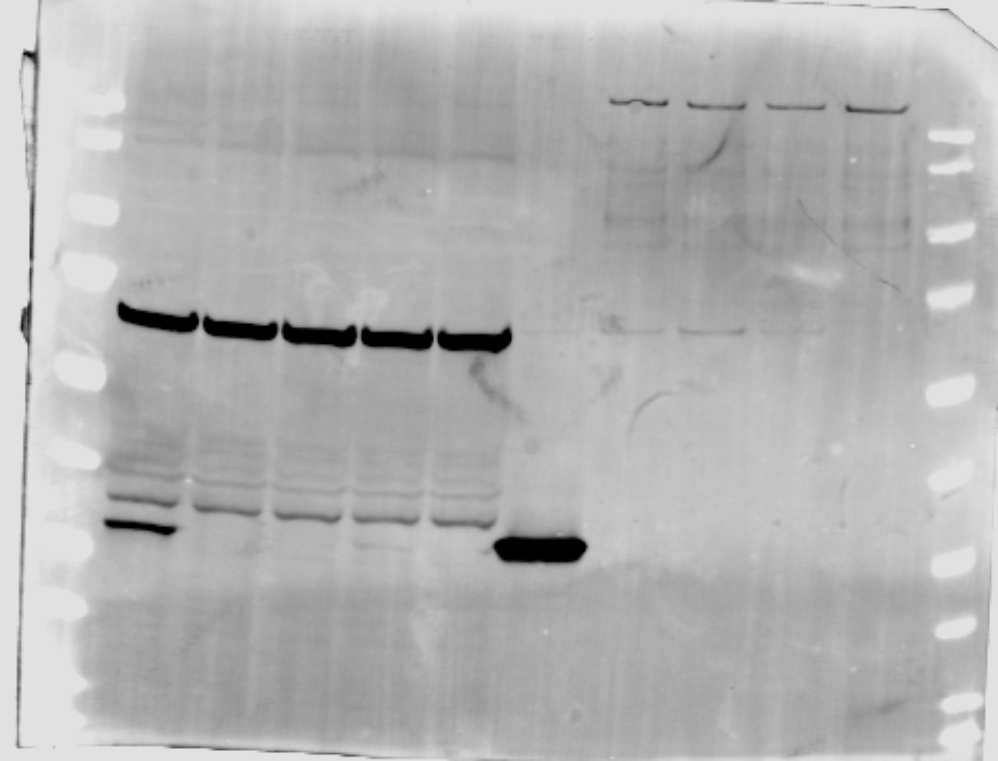

Supplement: Supplementary file 6 — Source Data Fig. 4 [file 44319_2023_43_MOESM6_ESM.zip › Figure 4/4E/UBAP2Lmutants_GFP.png]

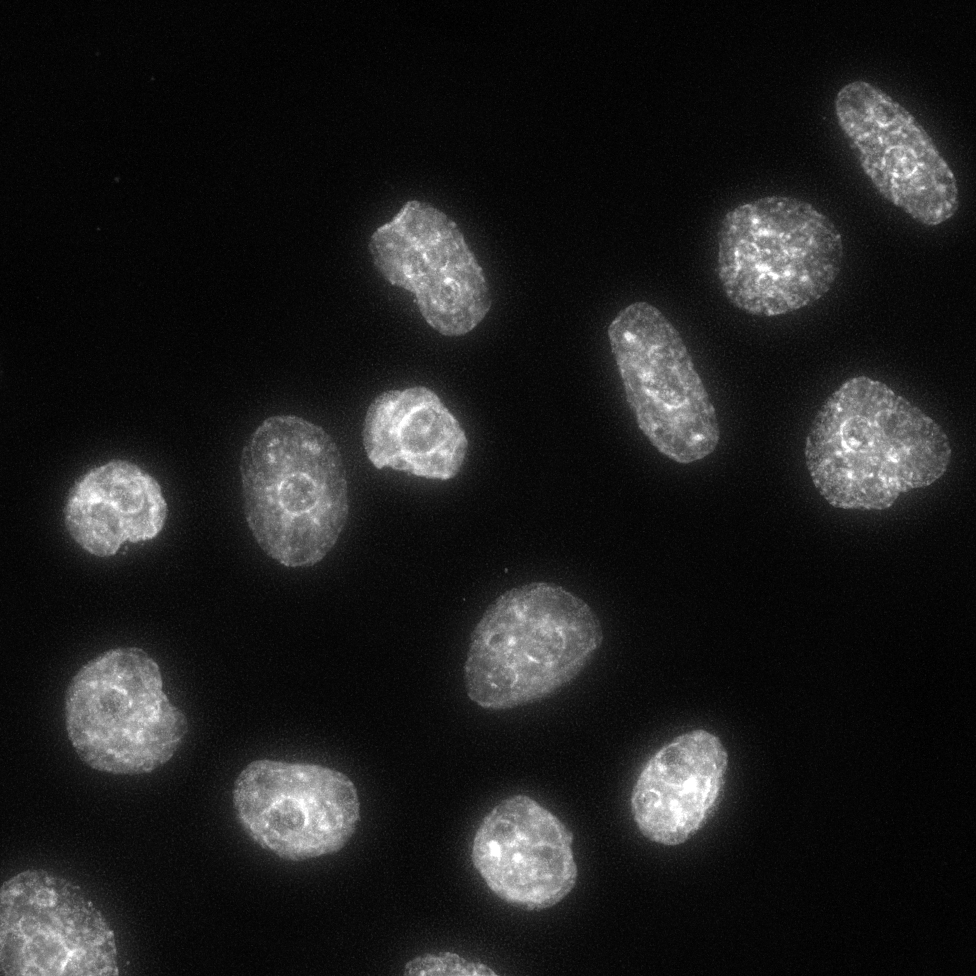

Supplement: Supplementary file 7 — Source Data Fig. 5 [file 44319_2023_43_MOESM7_ESM.zip › Figure 5/5A/Images/5A_image_MAX_frx_1169ctrl_3_R3D_D3D.tif]

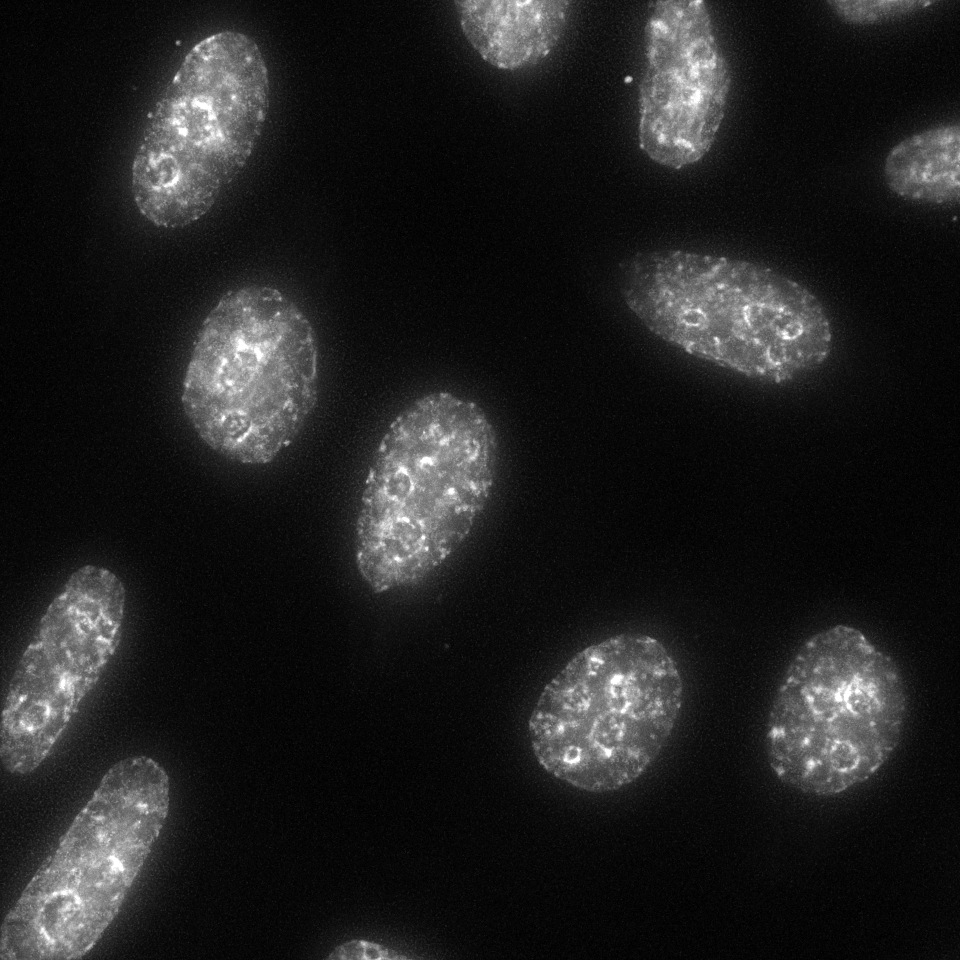

Supplement: Supplementary file 7 — Source Data Fig. 5 [file 44319_2023_43_MOESM7_ESM.zip › Figure 5/5A/Images/5A_image_MAX_fxr1_mut_20_R3D_D3D.tif]

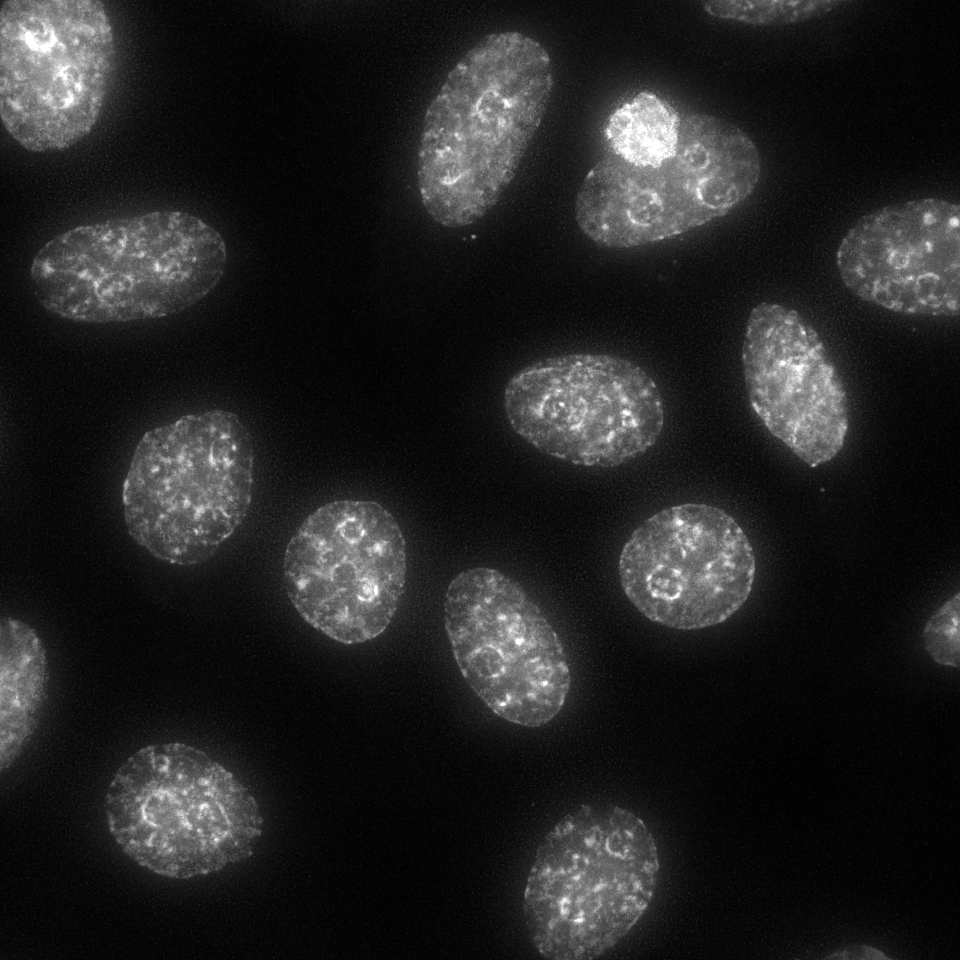

Supplement: Supplementary file 7 — Source Data Fig. 5 [file 44319_2023_43_MOESM7_ESM.zip › Figure 5/5A/Images/5B_image_MAX_fxr1_wt_6_R3D_D3D.tif]

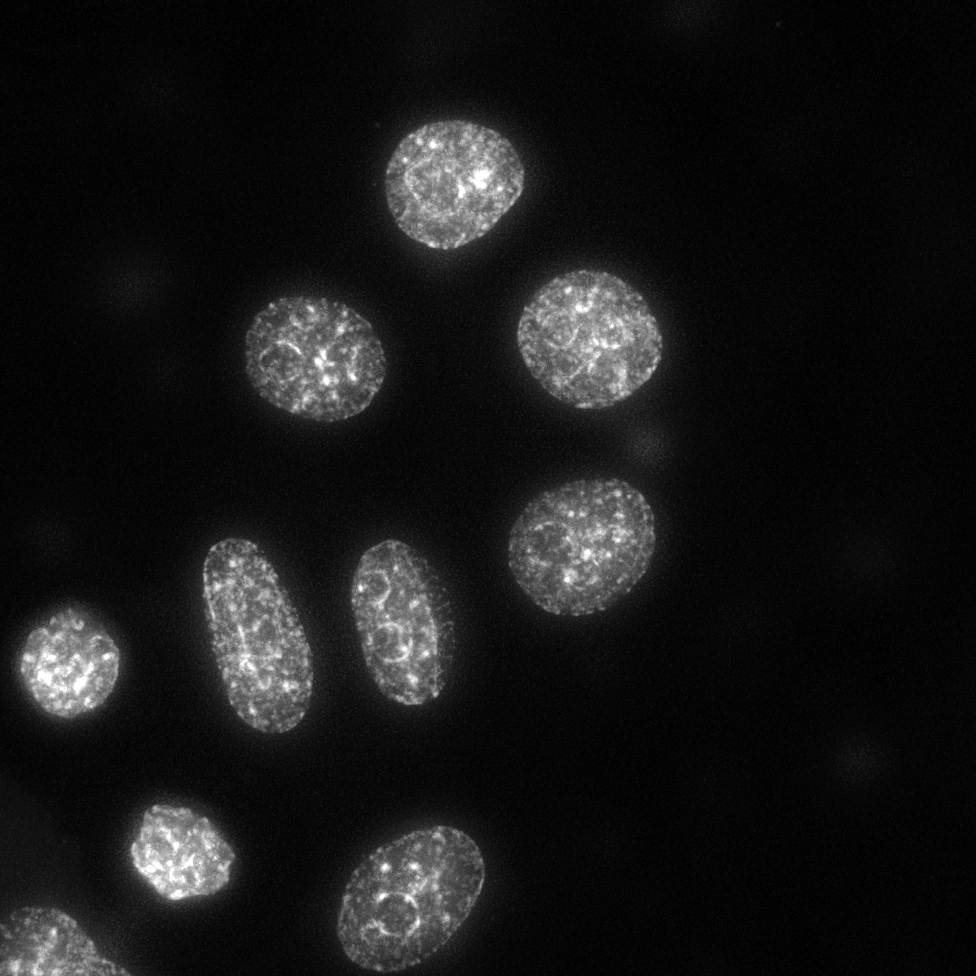

Supplement: Supplementary file 7 — Source Data Fig. 5 [file 44319_2023_43_MOESM7_ESM.zip › Figure 5/5B/Images/5B_image_MAX_G3_1169ctrl_3_R3D_D3D.tif]

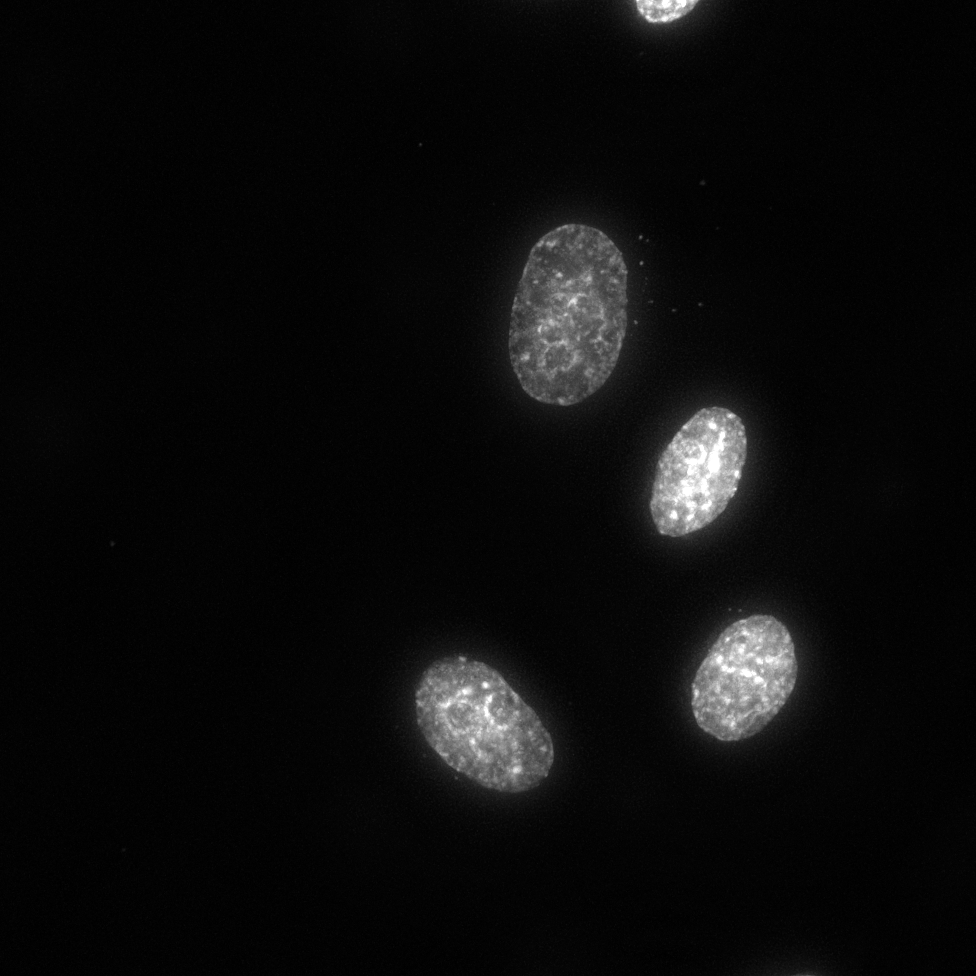

Supplement: Supplementary file 7 — Source Data Fig. 5 [file 44319_2023_43_MOESM7_ESM.zip › Figure 5/5B/Images/5B_image_MAX_G3_mut_4_R3D_D3D.tif]

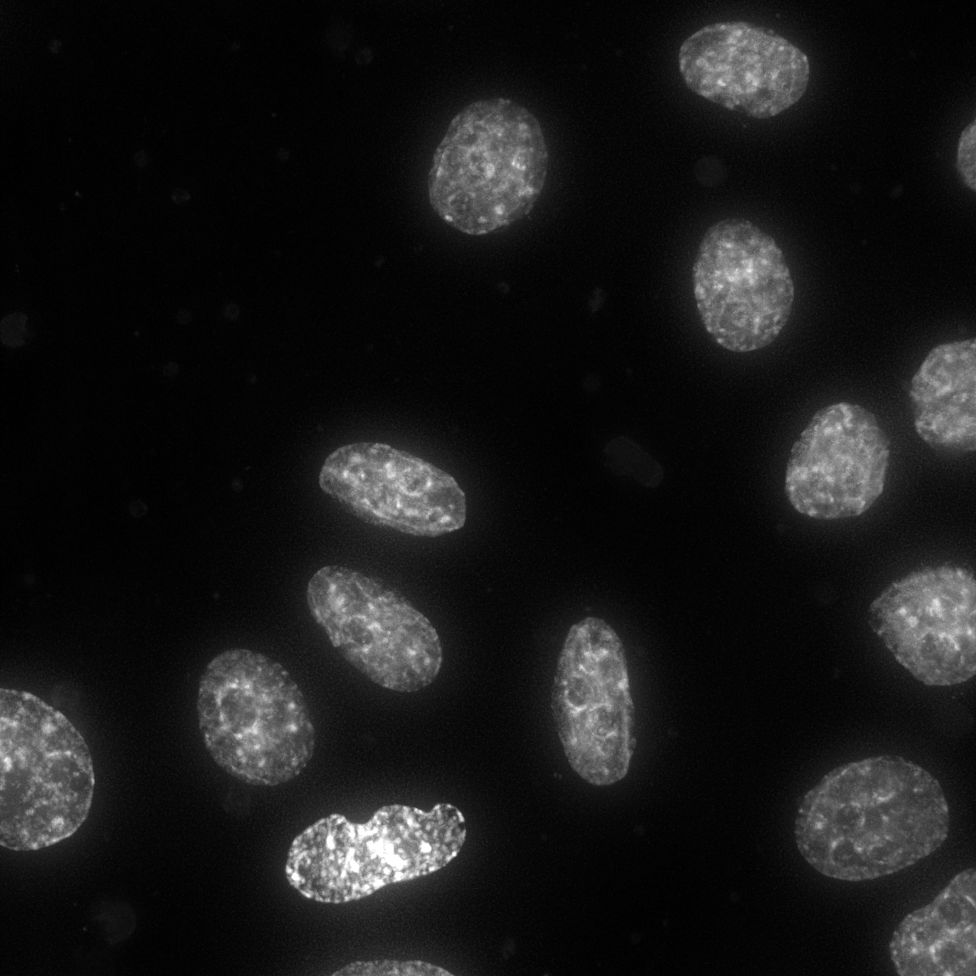

Supplement: Supplementary file 7 — Source Data Fig. 5 [file 44319_2023_43_MOESM7_ESM.zip › Figure 5/5B/Images/5B_image_MAX_G3_wt_2_R3D_D3D.tif]

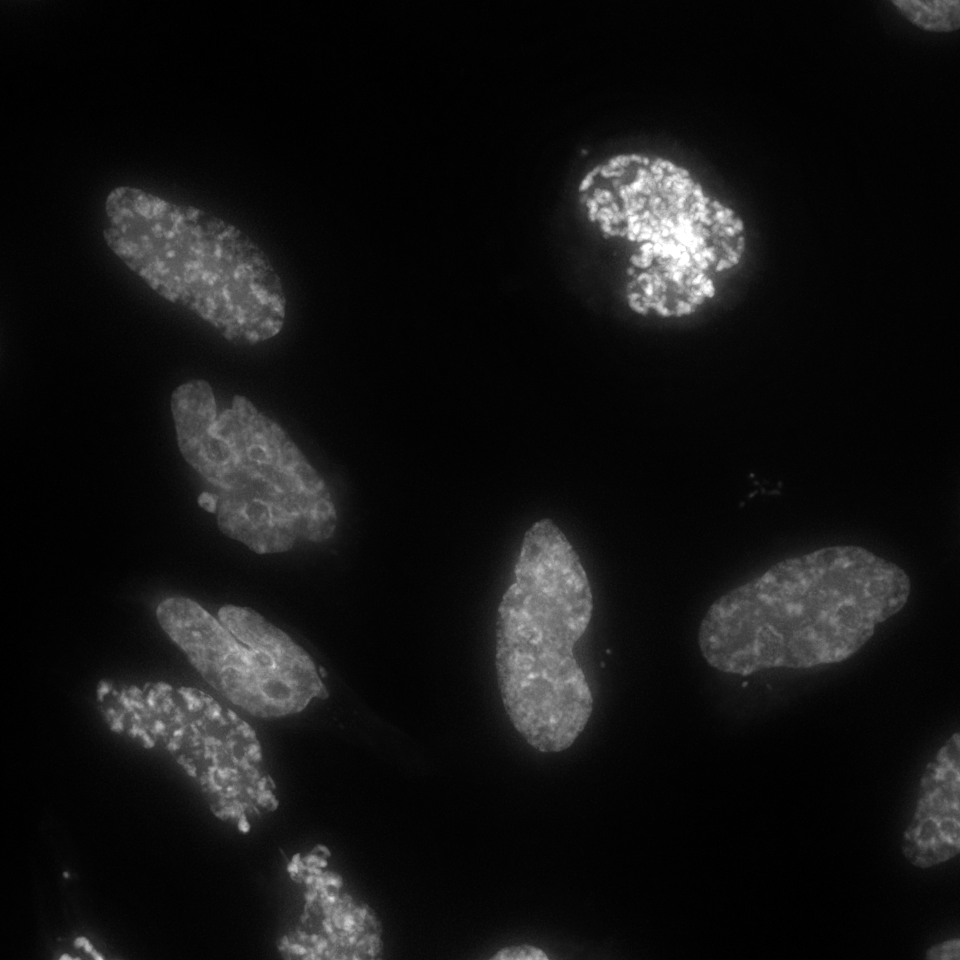

Supplement: Supplementary file 7 — Source Data Fig. 5 [file 44319_2023_43_MOESM7_ESM.zip › Figure 5/5C/5C_image_FXR1_MAX_FI_mut_2_R3D_D3D.tif]

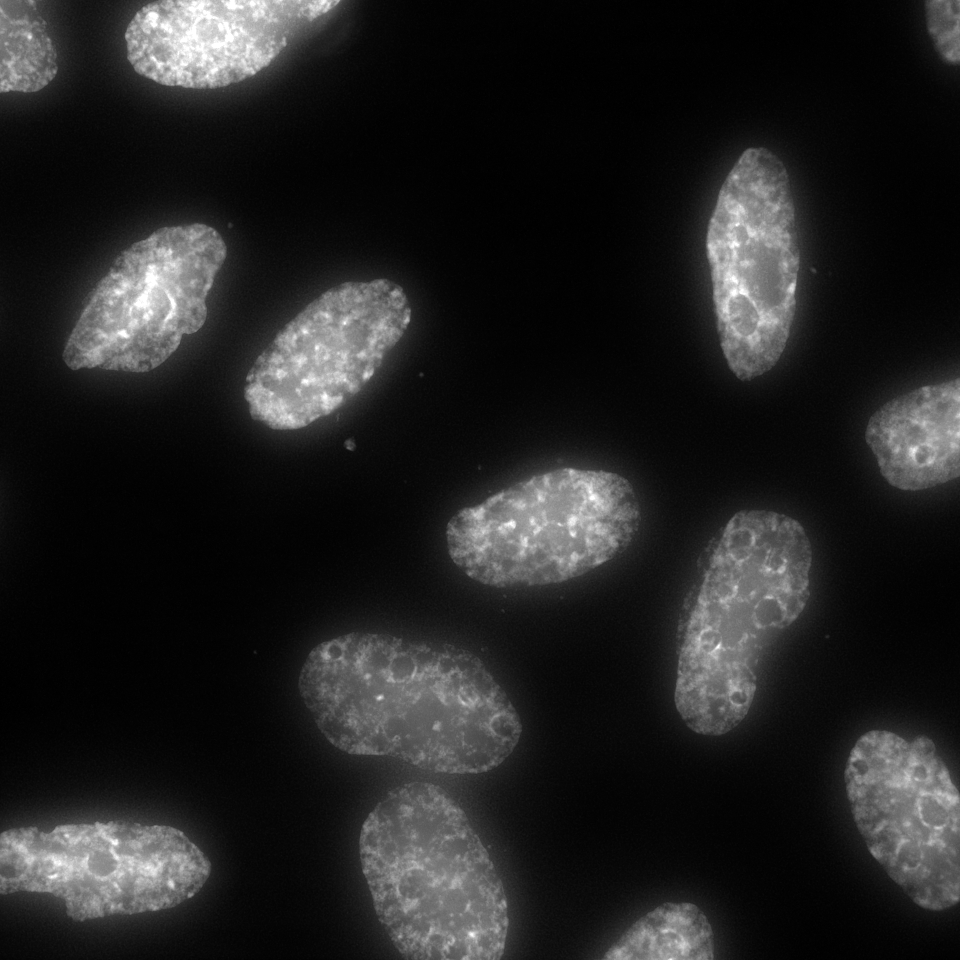

Supplement: Supplementary file 7 — Source Data Fig. 5 [file 44319_2023_43_MOESM7_ESM.zip › Figure 5/5C/5C_image_FXR1_MAX_wt_6_R3D_D3D.tif]

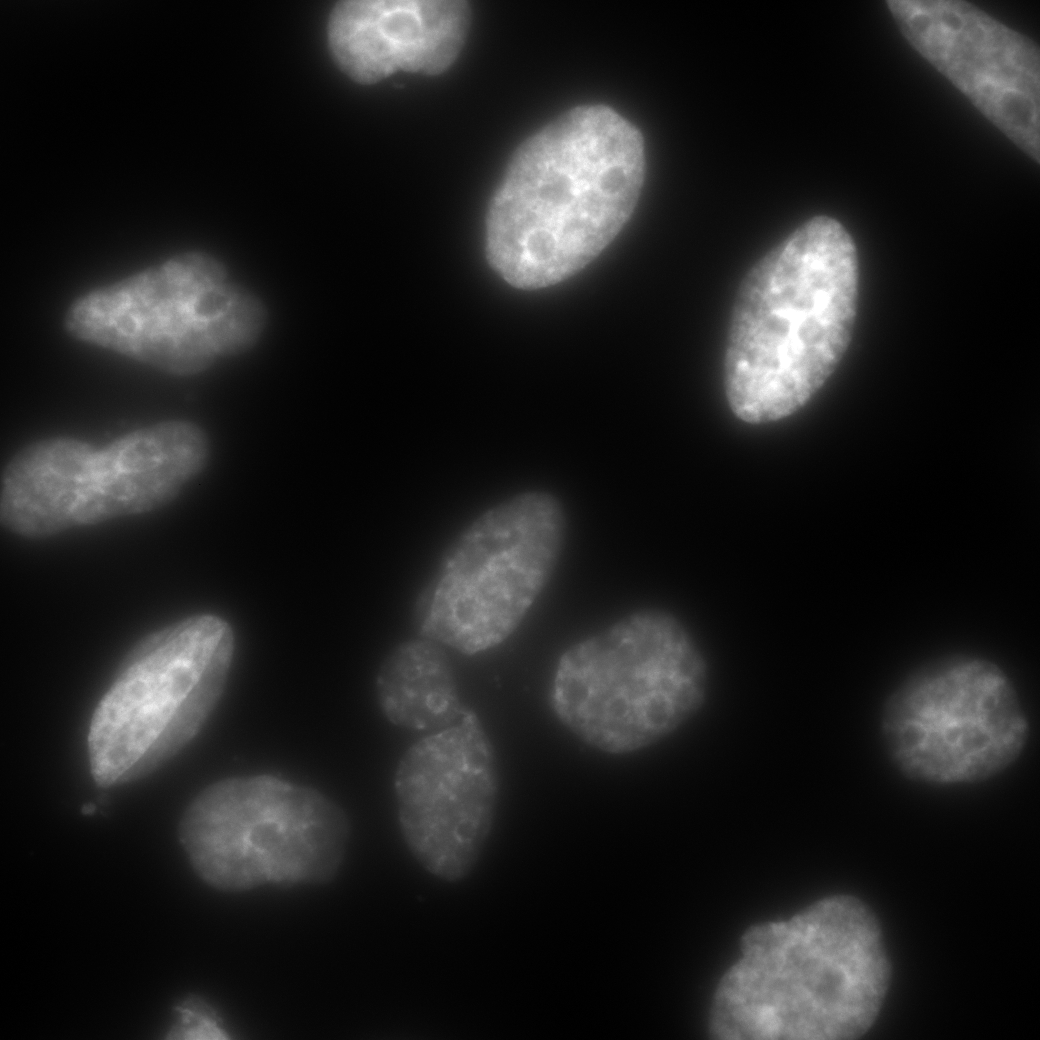

Supplement: Supplementary file 7 — Source Data Fig. 5 [file 44319_2023_43_MOESM7_ESM.zip › Figure 5/5C/5C_image_MAX_ctrl_ko_fxr1sgs_1_R3D.tif]

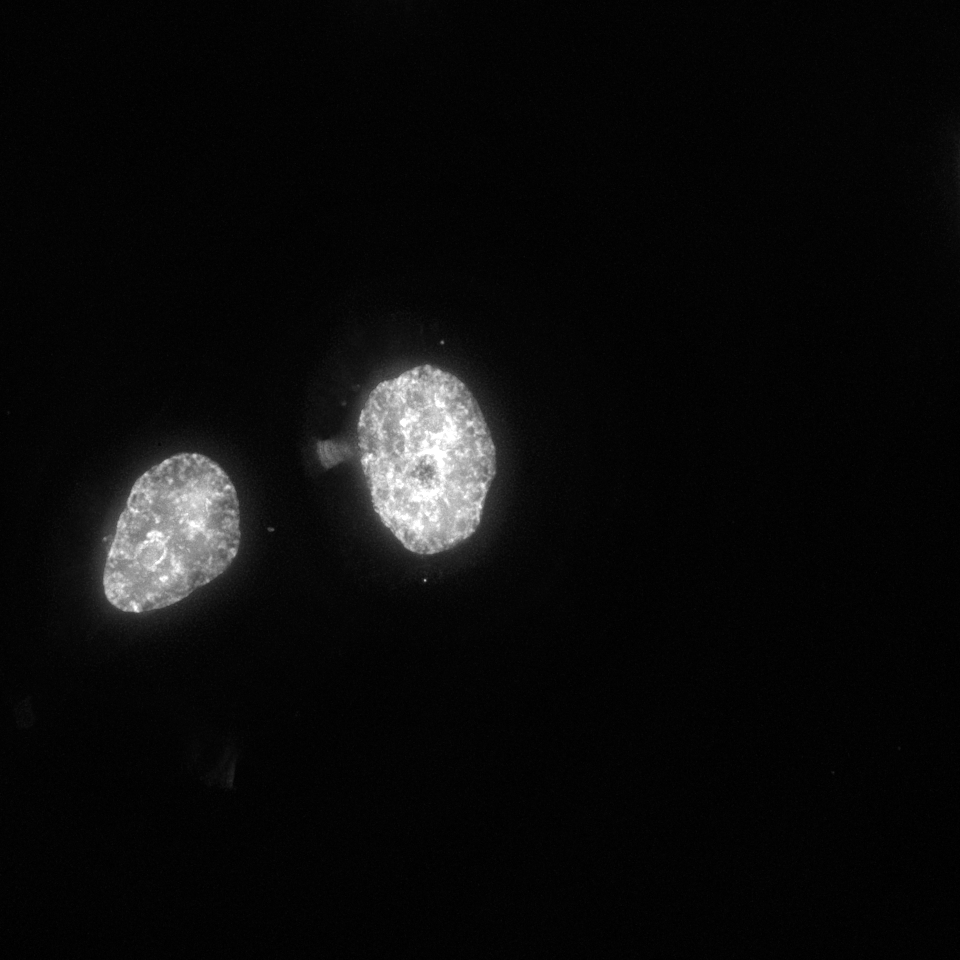

Supplement: Supplementary file 7 — Source Data Fig. 5 [file 44319_2023_43_MOESM7_ESM.zip › Figure 5/5C/5C_imageMAX_fxr1_ED_KK_3603ko_3_R3D_D3D.tif]

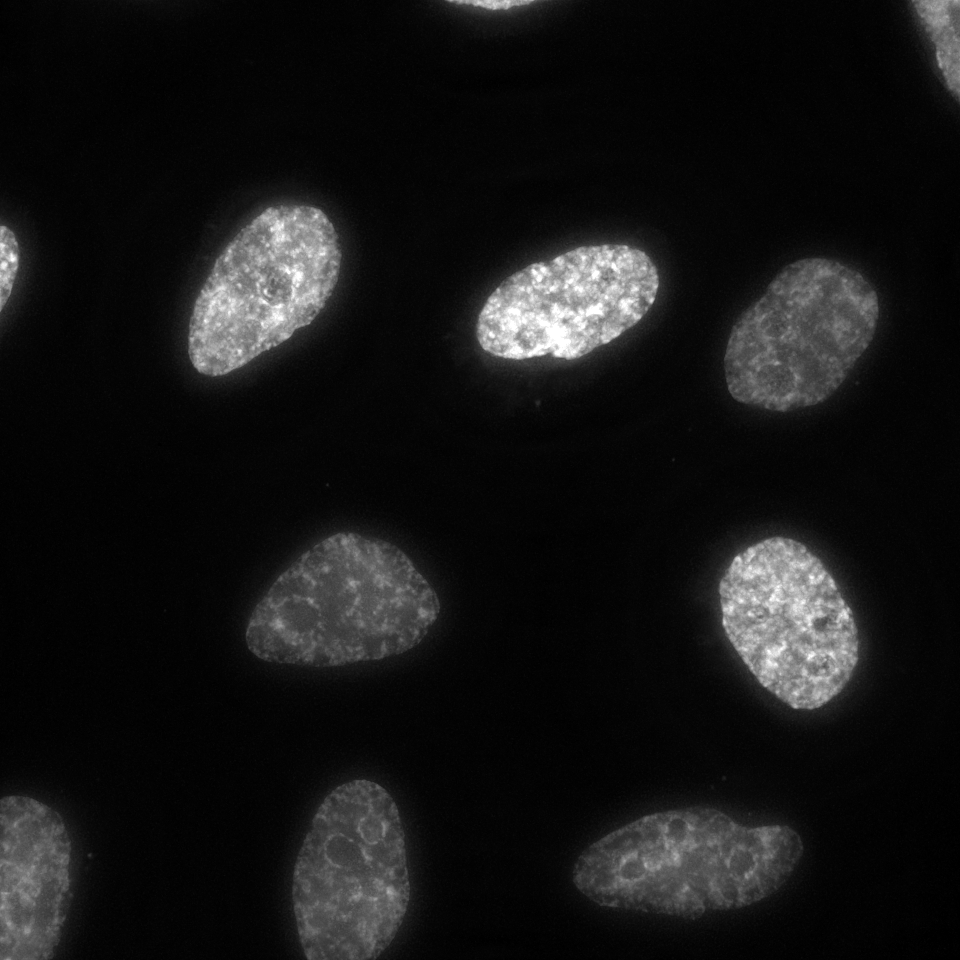

Supplement: Supplementary file 7 — Source Data Fig. 5 [file 44319_2023_43_MOESM7_ESM.zip › Figure 5/5D/5D_image_MAX_g3_YFPonlyko_5_R3D_D3D.tif]

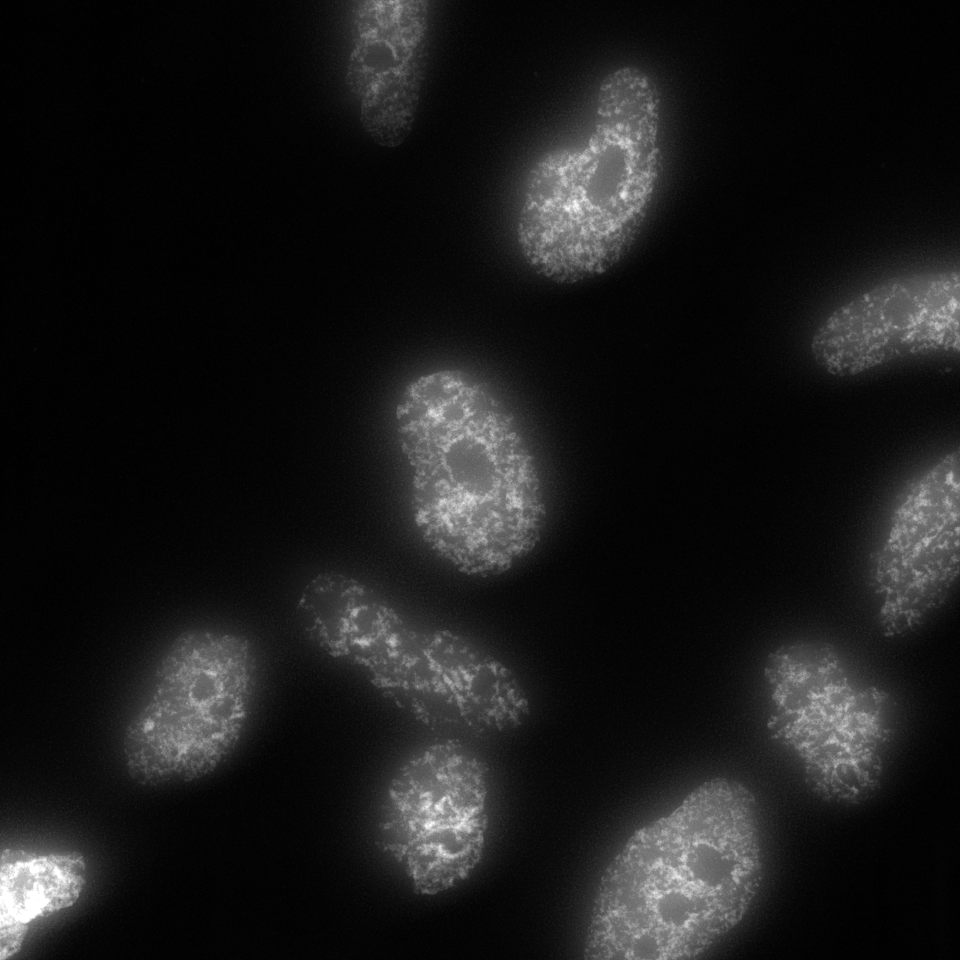

Supplement: Supplementary file 7 — Source Data Fig. 5 [file 44319_2023_43_MOESM7_ESM.zip › Figure 5/5D/5D_image_MAX_G3BP1foci_FImut_3_R3D_D3D.tif]

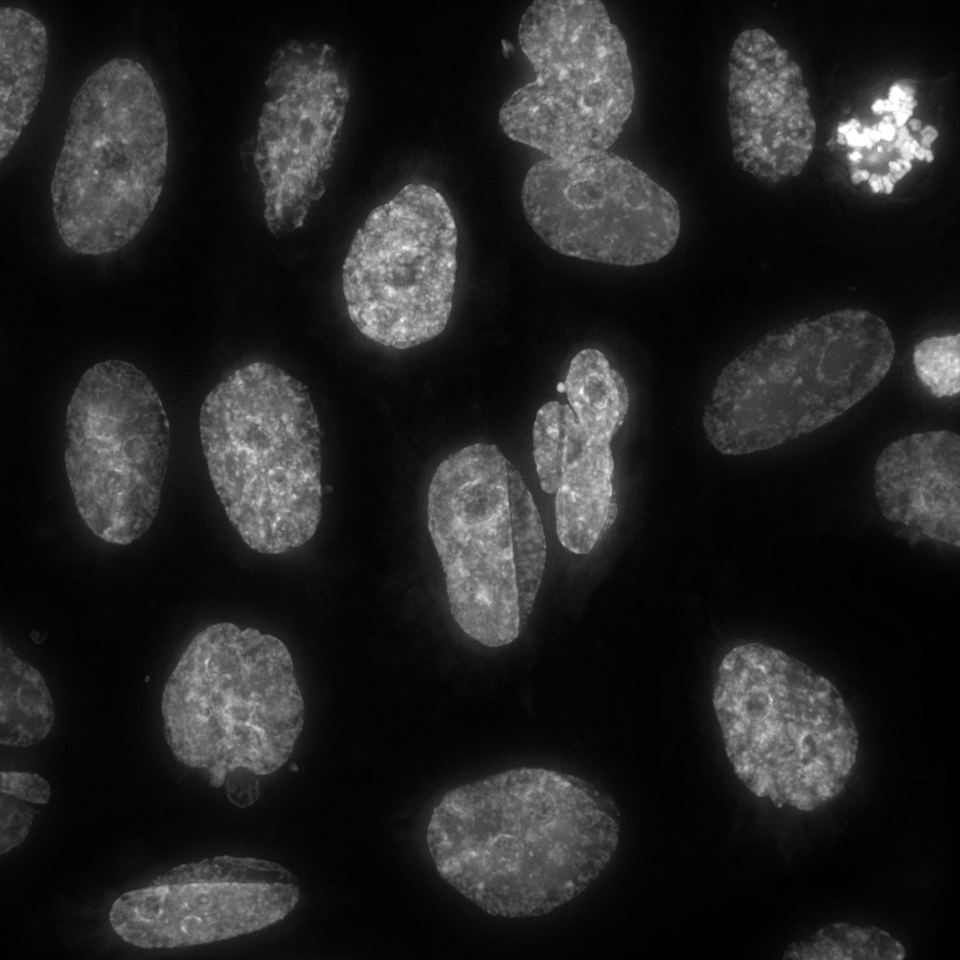

Supplement: Supplementary file 7 — Source Data Fig. 5 [file 44319_2023_43_MOESM7_ESM.zip › Figure 5/5D/5D_imageMAX_g3bp1_3603_EE_KKmutko_5_R3D_D3D.tif]

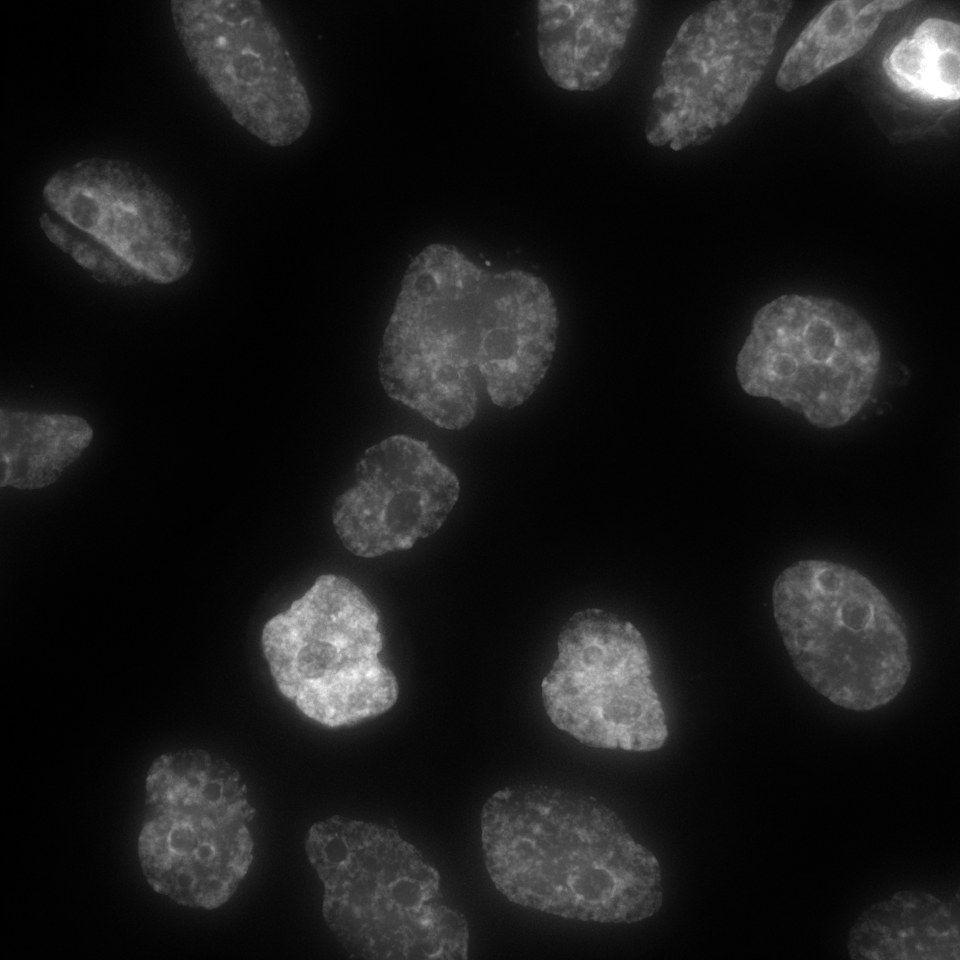

Supplement: Supplementary file 7 — Source Data Fig. 5 [file 44319_2023_43_MOESM7_ESM.zip › Figure 5/5D/5D_imageMAX_G3BP1foci_wt_5_R3D_D3D.tif]
