# Supplementary material for: The NSP3 protein of SARS-CoV-2 binds fragile X mental retardation proteins to disrupt UBAP2L interactions
Source: EMBO Rep. 2024 Jan 2;25(2):25. doi: 10.1038/s44319-023-00043-z (PMC10897489; doi:10.1038/s44319-023-00043-z)
Supplement: Supplementary file 4 — Source Data Fig. 2 [file 44319_2023_43_MOESM4_ESM.zip › Figure 2/2A/2A.rtf]

2AGolden Syrian hamsters were infected with 105 plaque forming units (PFU) of WT SARS-CoV-2 (n=15), NSP3 mutants (n=15), or mock (PBS, n=15) and monitored for weight loss and signs of disease over a 7 day time course.  
