# Supplementary material for: The NSP3 protein of SARS-CoV-2 binds fragile X mental retardation proteins to disrupt UBAP2L interactions
Source: EMBO Rep. 2024 Jan 2;25(2):25. doi: 10.1038/s44319-023-00043-z (PMC10897489; doi:10.1038/s44319-023-00043-z)
Supplement: Supplementary file 4 — Source Data Fig. 2 [file 44319_2023_43_MOESM4_ESM.zip › Figure 2/2B/2B.rtf]

2B At days 2, 4, and 7 post infection, hamsters (n=5 individual hamsters) were nasal washed and subsequently euthanized and tissue collected to assay viral titers from lung 
