# Supplementary material for: The NSP3 protein of SARS-CoV-2 binds fragile X mental retardation proteins to disrupt UBAP2L interactions
Source: EMBO Rep. 2024 Jan 2;25(2):25. doi: 10.1038/s44319-023-00043-z (PMC10897489; doi:10.1038/s44319-023-00043-z)
Supplement: Supplementary file 4 — Source Data Fig. 2 [file 44319_2023_43_MOESM4_ESM.zip › Figure 2/2D/2D.rtf]

2DRepresentative lung tissue sections stained for viral antigen (nucleocapsid) at day 2 from WT, NSP3 mut 1 and mut 2 infected animals.
