# Supplementary material for: The NSP3 protein of SARS-CoV-2 binds fragile X mental retardation proteins to disrupt UBAP2L interactions
Source: EMBO Rep. 2024 Jan 2;25(2):25. doi: 10.1038/s44319-023-00043-z (PMC10897489; doi:10.1038/s44319-023-00043-z)
Supplement: Supplementary file 4 — Source Data Fig. 2 [file 44319_2023_43_MOESM4_ESM.zip › Figure 2/2F/2F.rtf]

2FAntigen staining was scored on a 4 points scale for the airway for WT and NSP3 mutants on days However,  at day 4, these trends reversed with both NSP3 mutants showing more antigen staining than2, 4, and 7 in a blinded manner. Each data point representative of the average score from two lung section from each hamsters in the group (n=5 individual hamsters). Statistical analysis measured by two-tailed Student’s t-test: ***p<0.001, **p<0.01. H) 
