# Supplementary material for: The NSP3 protein of SARS-CoV-2 binds fragile X mental retardation proteins to disrupt UBAP2L interactions
Source: EMBO Rep. 2024 Jan 2;25(2):25. doi: 10.1038/s44319-023-00043-z (PMC10897489; doi:10.1038/s44319-023-00043-z)
Supplement: Supplementary file 5 — Source Data Fig. 3 [file 44319_2023_43_MOESM5_ESM.zip › Figure 3/3C/3C.rtf]

3CMembranes were spotted with peptides and incubated with FXR1 or BSA (for control) protein and analysed by western blot for FXR1 binding. 
