# Supplementary material for: The NSP3 protein of SARS-CoV-2 binds fragile X mental retardation proteins to disrupt UBAP2L interactions
Source: EMBO Rep. 2024 Jan 2;25(2):25. doi: 10.1038/s44319-023-00043-z (PMC10897489; doi:10.1038/s44319-023-00043-z)
Supplement: Supplementary file 6 — Source Data Fig. 4 [file 44319_2023_43_MOESM6_ESM.zip › Figure 4/4G/4G.rtf]

4GCompetition between NSP3 peptide and UBAP2L peptide for binding to FXR1 215-360. The black trace is NSP3 binding to FXR1 while the red trace is NSP3 binding to FXR1 preincubated with UBAP2L peptide. 
