# Supplementary material for: The NSP3 protein of SARS-CoV-2 binds fragile X mental retardation proteins to disrupt UBAP2L interactions
Source: EMBO Rep. 2024 Jan 2;25(2):25. doi: 10.1038/s44319-023-00043-z (PMC10897489; doi:10.1038/s44319-023-00043-z)
Supplement: Supplementary file 7 — Source Data Fig. 5 [file 44319_2023_43_MOESM7_ESM.zip › Figure 5/5C/IF_images_generation and analysis.rtf]

Images were acquired on a DelteVision Elite microscope and deconvoluted via the worX software. Fiji was used to generate a Z-stack projection with MAX setting. Foci were identified/measured via the find maxima tool in Fiji.
