# Supplementary material for: The NSP3 protein of SARS-CoV-2 binds fragile X mental retardation proteins to disrupt UBAP2L interactions
Source: EMBO Rep. 2024 Jan 2;25(2):25. doi: 10.1038/s44319-023-00043-z (PMC10897489; doi:10.1038/s44319-023-00043-z)
Supplement: Supplementary file 9 — Expanded View Figures [file 44319_2023_43_MOESM9_ESM.pdf]

Expanded View Figures

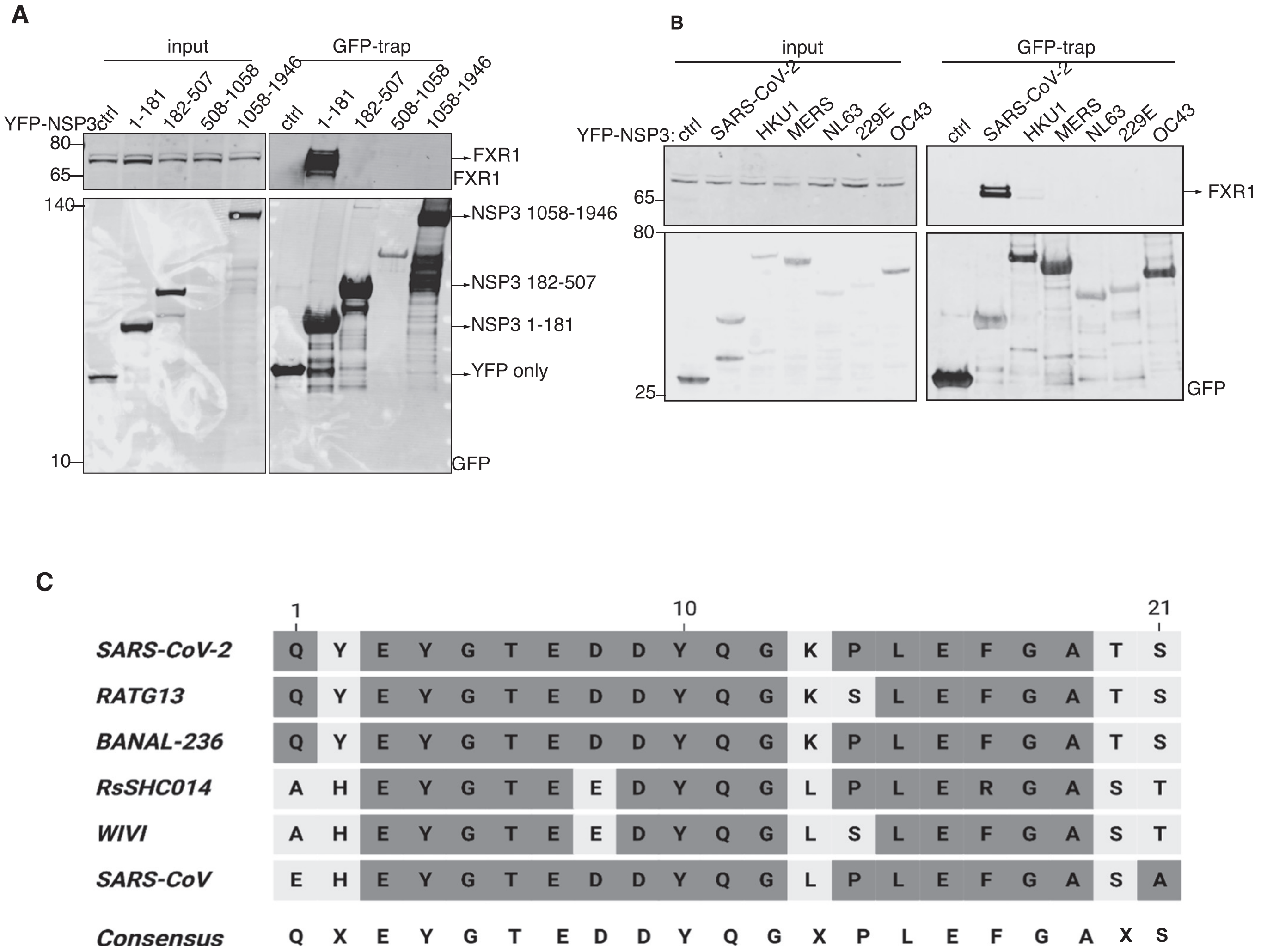

Figure EV1. Analysis of FMRP-NSP3 interaction.

(**A**) The indicated NSP3 fragments fused to YFP was expressed and purified from HeLa cells and binding to FXR1 monitored. Representative of two biological replicates. (**B**) A panel of NSP3 N-terminal fragments from different coronaviruses were expressed and purified from HeLa cells and binding to FXR1 determined by western blotting. Representative of two biological replicates. (**C**) Alignment of the NSP3 sequence binding to FMRPs from different coronaviruses.

 © The Author(s)

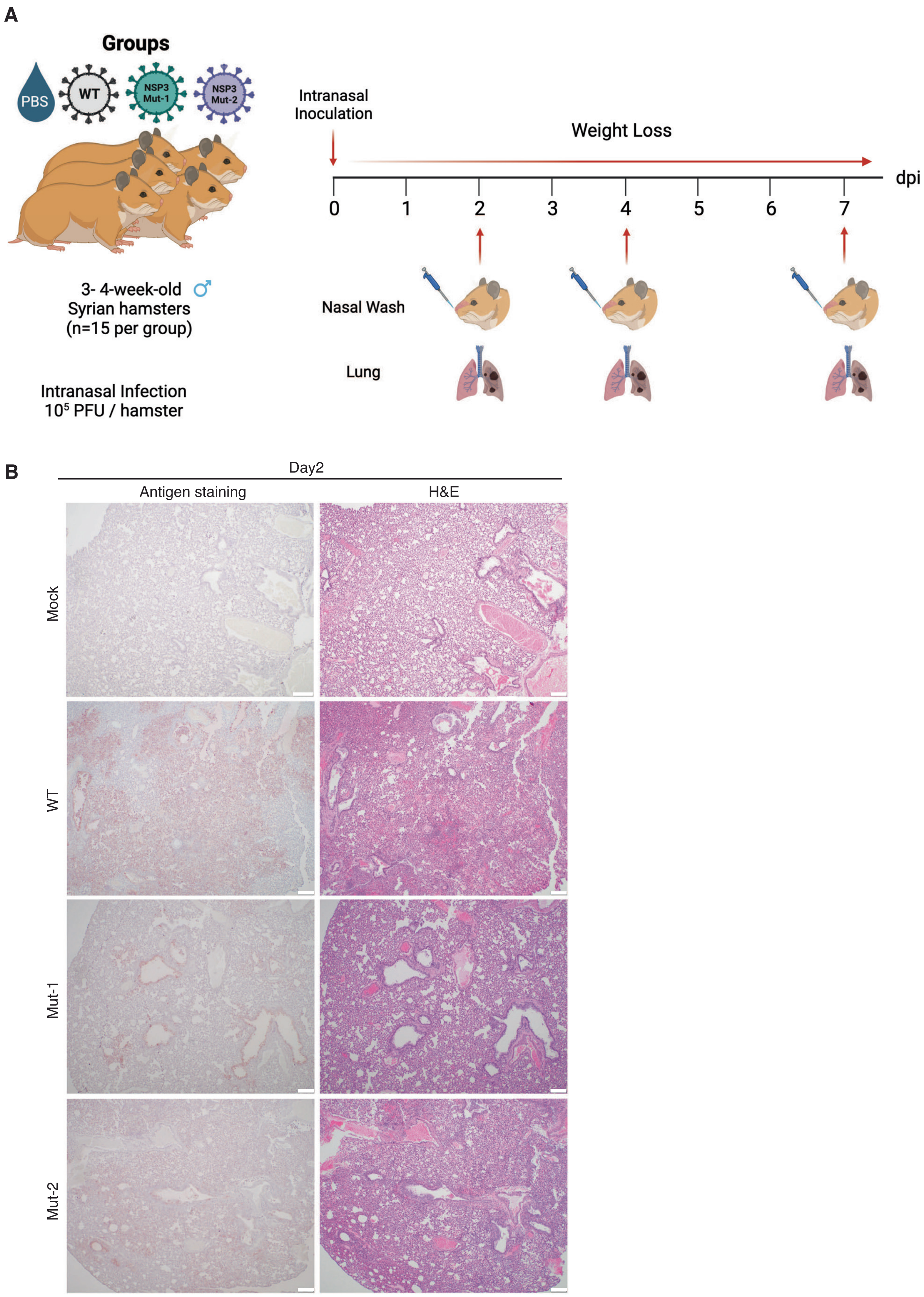
A
Groups
PBS
WT
NSP3 Mut-1
NSP3 Mut-2
3- 4-week-old ♂
Syrian hamsters
(n=15 per group)
Intranasal Infection
10^5 PFU / hamster
Intranasal Inoculation
Weight Loss
dpi
0 1 2 3 4 5 6 7
Nasal Wash
Lung
B
Day2
Antigen staining
H&E
Mock
WT
Mut-1
Mut-2

Figure EV2. Histopathology of hamster infected with WT or NSP3 Mutants.

(**A**) Schematic of in vivo experiment (generated with BioRender). (**B**) H&E and viral antigen (nucleocapsid) immunohistochemical staining of lung of hamsters infected mock (PBS) or with 10^5 pfu of WT, NSP3 Mut-1, or NSP3 Mut-2 SARS-CoV-2 at 2 days post infection. WT infection shows extensive viral infection and damage; both NSP3 mutants have focal disease and less damage. No damage observed in mock infected samples. Images from representative section from a single hamster in each group (mock, WT, NSP3 Mut-1, & NSP3 mut-2). For larger magnification see Fig. 2. Data information: Scale bar of 200 μm is indicated in the lower right corner.

© The Author(s)

A

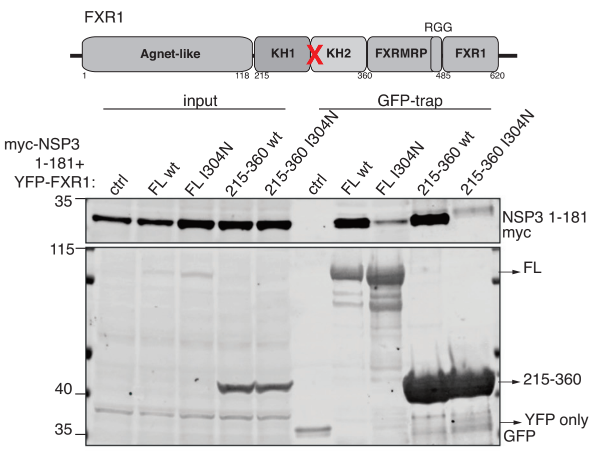

B

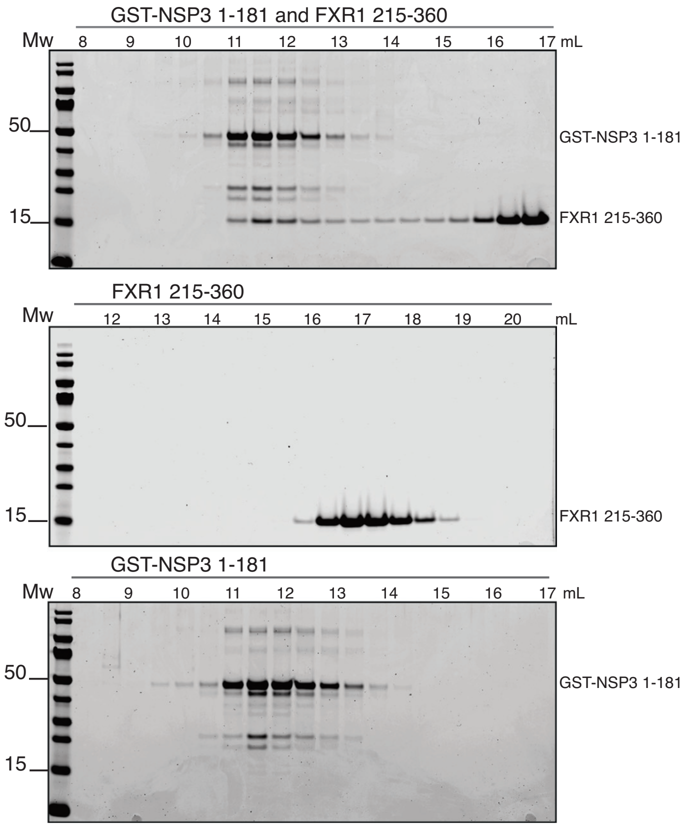

C

Binding affinities of GST-NSP3 1-181

Protein	K_D (μM)
$FXR1_{1-122}$	NB
$FXR1_{212-289}$	NB
$FXR1_{215-360}$	2,3

Binding affinities of $P1234_{1807-1840}$ alphavirus

Protein	K_D (μM)
$FXR1_{1-122}$	19,4
$FXR1_{212-289}$	NB
$FXR1_{215-360}$	NB

D

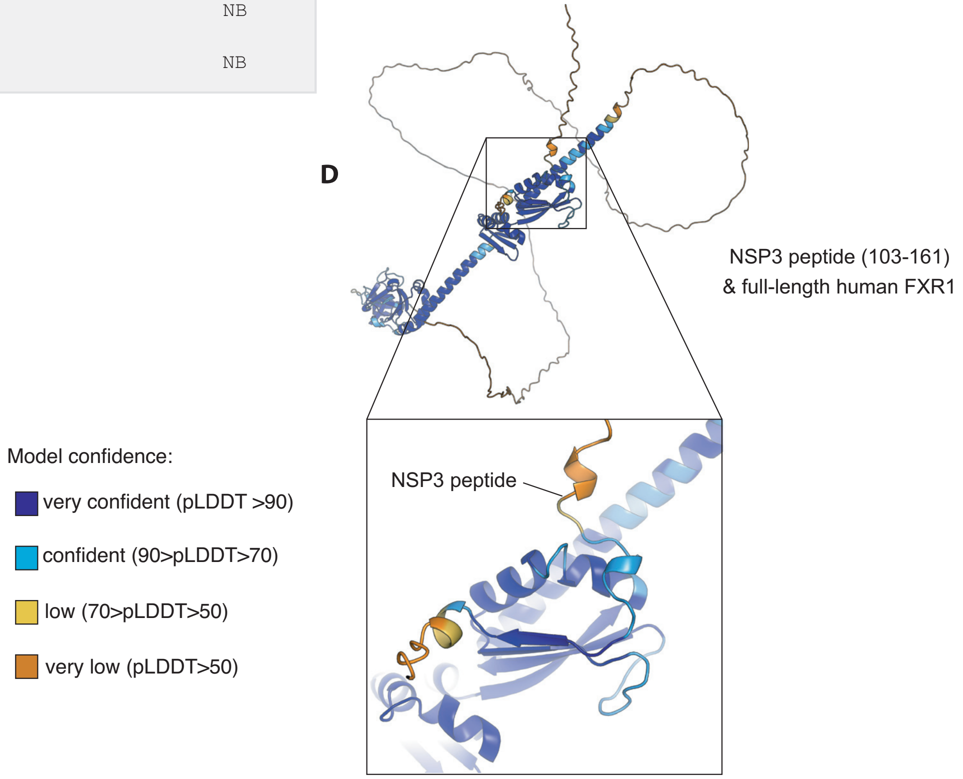

Figure EV3. A direct interaction between NSP3 and FXR1.

(**A**) The indicated FXR1 YFP constructs were co-expressed with myc-NSP3 1–181 in HeLa cells and affinity-purified using YFP affinity beads. The binding to NSP3 was monitored by probing for myc. Representative of two biological replicates. (**B**) Size-exclusion chromatography of GST-NSP3 WT 1–181, FXR1 215–360 either alone or in combination. The elution volume is indicated on top and Coomasie stained gels of fractions shown. Representative of two biological replicates. (**C**) Table of ITC values obtained for the indicated FXR1 fragments binding to GST-NSP3 1–181 or the FXR1 binding peptide from old alphaviruses ($n = 1$). (**D**) Confidence plots of AlphaFold model of NSP3 peptide binding to full-length FXR1.

© The Author(s)

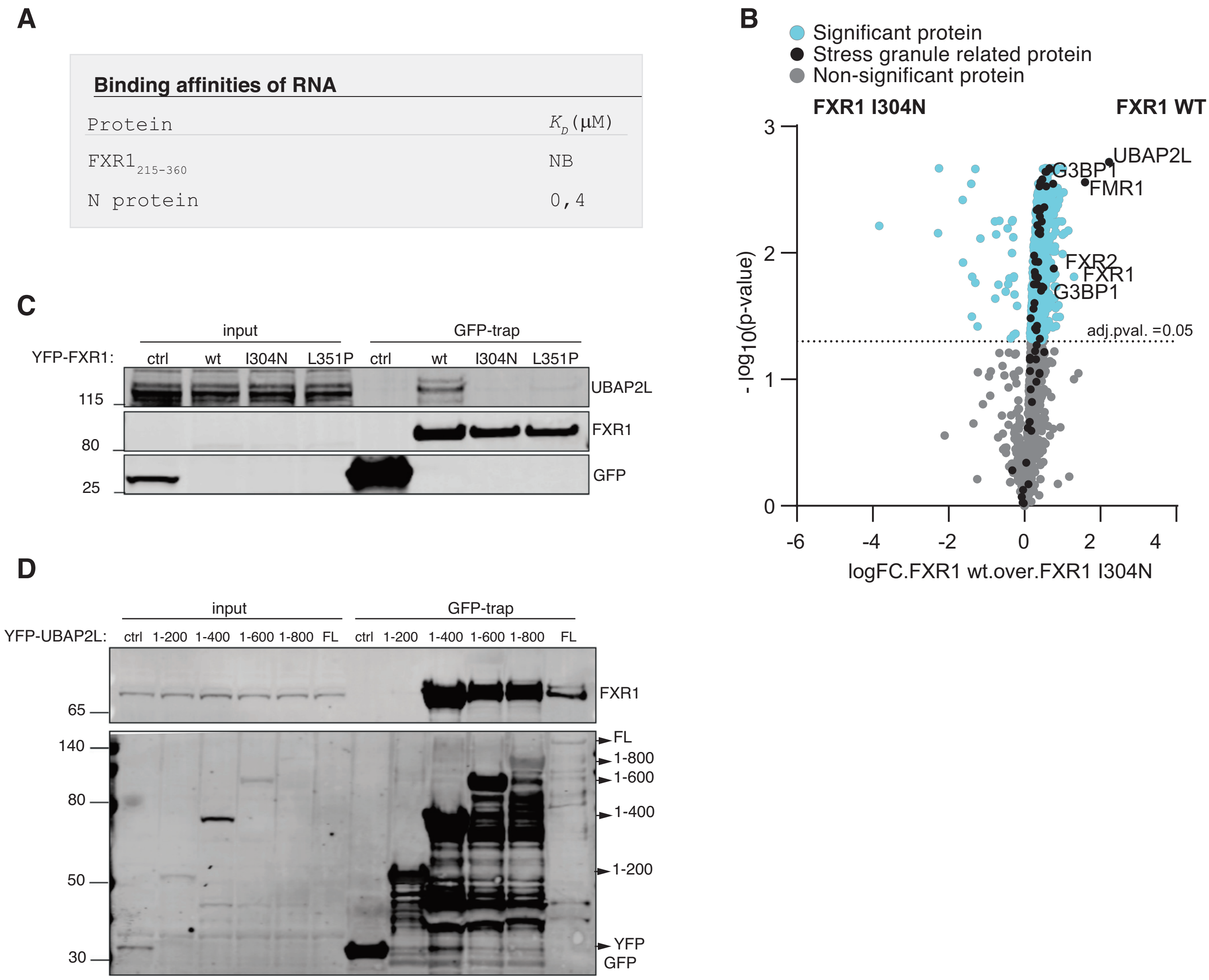

Figure EV4. Interaction of FXR1 to UBAP2L.

(**A**) ITC measurements of a reported RNA binding to FXR1. Binding to FXR1 215–360 was monitored and as a control the SARS-CoV-2 N protein ($n = 1$). (**B**) Mass spectrometry analysis of the interactomes of affinity-purified YFP-tagged FXR1 WT and FXR1 I304N. Proteins specifically binding to FXR1 WT indicated in the volcano plot. Data from 4 technical repeats. (**C**) The indicated YFP-tagged FXR1 proteins were expressed and purified from HeLa cells and binding to UBAP2L determined by western blot. Representative of 2 biological replicates. (**D**) A panel of YFP-UBAP2L constructs were expressed and purified from HeLa cells and binding to FXR1 determined. Representative of 2 biological replicates. Data information: In (**B**) a two-sided unpaired *t* test was used for statistical analysis.

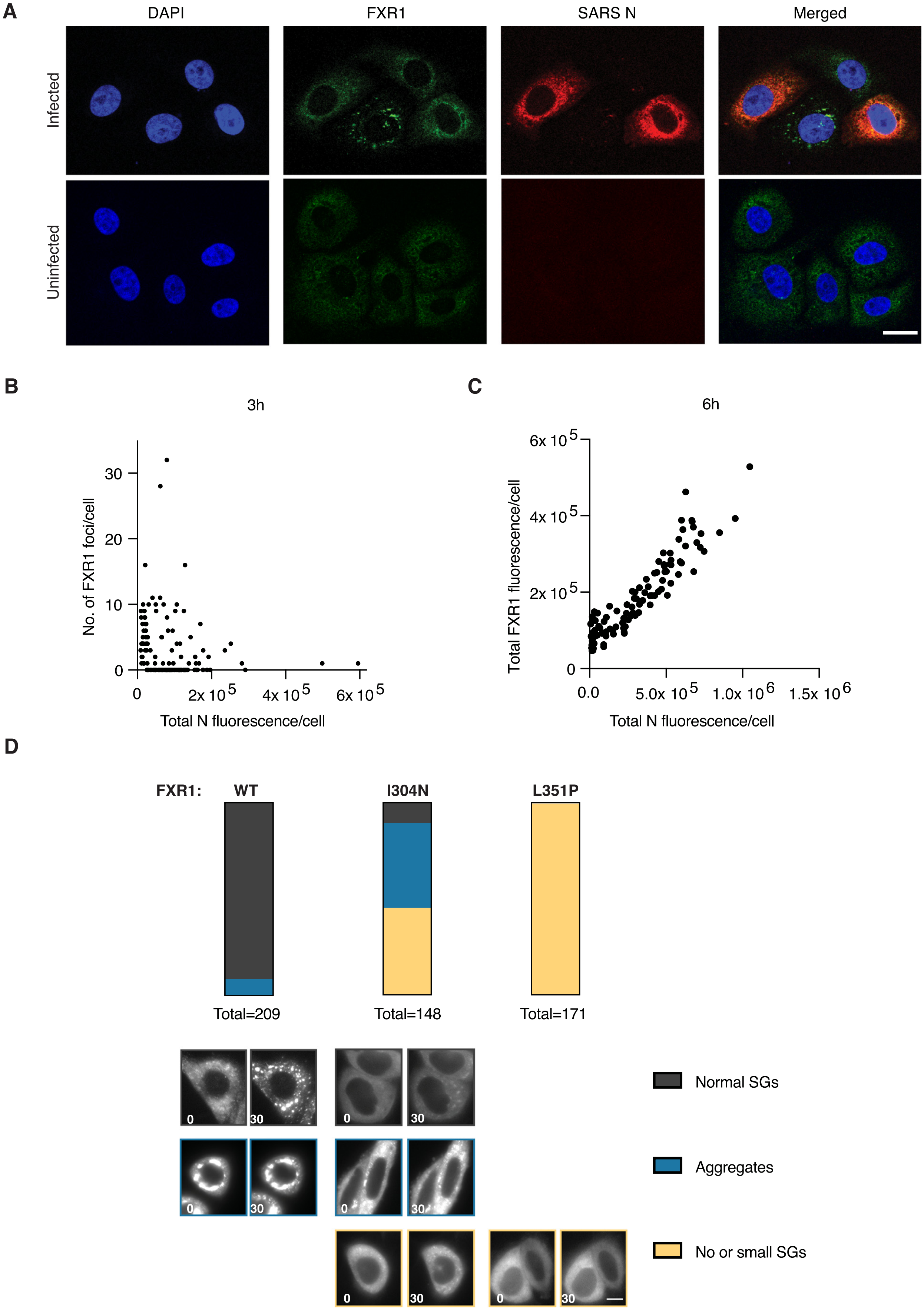
A
DAPI
FXR1
SARS N
Merged
Infected
Uninfected
B
3h
No. of FXR1 foci/cell
Total N fluorescence/cell
C
6h
Total FXR1 fluorescence/cell
Total N fluorescence/cell
D
FXR1:
WT
I304N
L351P
Total=209
Total=148
Total=171
Normal SGs
Aggregates
No or small SGs

 © The Author(s)

Figure EV5. Analysis of FXR1 localization to stress granules.

(**A**) VeroE6 cells infected with SARS-CoV-2 or uninfected were fixed and stained for FXR1 and the viral N protein. Representative images shown from one experiment. (**B**) The number of FXR1 foci in infected cells was determined and correlated with total level of N protein. (**C**) The total level of FXR1 was determined in infected cells and plotted against total levels of N. (**D**) YFP-tagged FXR1 proteins were expressed in HeLa cells and filmed by live-cell microscopy. Stress granule formation was induced by arsenite and 30 min after addition the localization and morphology of FXR1 foci was monitored. Phenotypes are plotted as percentage. Scores of two individual experiments are shown. The total number of cells analyzed per condition are indicated. Representative images are shown. Data information: In (**A**) scale bar is 20 μm and in (**D**) it is 10 μm.
